# Improved Myocardial Sodium Quantification at 7 T Using Interleaved ^23^Na/ ^1^H pTx MRI With Motion and Anatomy‐Based B_1_ Correction

**DOI:** 10.1002/mrm.70342

**Published:** 2026-03-16

**Authors:** Laurent Ruck, Nico Egger, Benedikt Zobler, Judith Schirmer, Sophia Nagelstraßer, Andreas Bitz, Tanja Platt, Simon Konstandin, Christoph Kopp, Michael Uder, Armin Michael Nagel

**Affiliations:** ^1^ Institute of Radiology, University Hospital Erlangen, Friedrich‐Alexander‐Universität Erlangen‐Nürnberg (FAU) Erlangen Germany; ^2^ Department of Nephrology and Hypertension Friedrich‐Alexander‐Universität Erlangen‐Nürnberg (FAU) Erlangen Germany; ^3^ Electrical Engineering and Information Technology University of Applied Sciences – FH Aachen Aachen Germany; ^4^ Medical Physics in Radiology German Cancer Research Center (DKFZ) Heidelberg Heidelberg Germany; ^5^ Fraunhofer Institute for Digital Medicine MEVIS Bremen Germany

**Keywords:** cardiac sodium (^23^Na) MRI, interleaved dual‐nuclear MRI, respiratory and cardiac motion correction, transmit and receive (B_1_) correction, ultrahigh field strength (7 T)

## Abstract

**Purpose:**

To improve accuracy and repeatability of myocardial ^23^Na quantification in cardiac MRI at 7 T by combining retrospective respiratory and cardiac motion correction with a novel anatomy‐based B_1_ bias field correction.

**Methods:**

In this study, a dual‐nuclear interleaved ^23^Na/^1^H MRI sequence at 7 T was applied. Here, ^1^H MR data enabled automated segmentation of myocardium and blood pool via nnUNet and facilitated respiratory and cardiac motion correction for both contrasts using ^1^H navigators. For ^23^Na MRI, a novel anatomy‐based B_1_ bias field correction was developed, estimating the transmit and receive (B_1_) field bias from ^1^H‐derived segmentations and synthetic ^23^Na images. All correction methods were validated through realistic simulations and in vivo studies. Repeatability was assessed in 10 healthy subjects, and the proposed B_1_ correction was compared to phantom‐based methods.

**Results:**

Respiratory and cardiac motion correction reduced quantification errors and improved SNR compared to conventional gating. The anatomy‐based B_1_ correction effectively mitigated errors caused by B_1_ inhomogeneities and outperformed phantom‐based methods in terms of repeatability. The measured apparent tissue sodium concentration of the myocardium after all corrections (49.5 ± 4.7 mM) was consistent with literature values. Combined motion and B_1_ corrections improved repeatability, reducing the coefficient of repeatability from 4.9 mM (11.2%) without corrections to 2.0 mM (4.0%) after corrections.

**Conclusion:**

The combination of retrospective motion correction and anatomy‐based B_1_ bias field correction enables repeatable myocardial sodium quantification at 7 T. The interleaved acquisition facilitates robust segmentation and efficient use of ^1^H MRI for motion and B_1_ correction, providing a scalable framework for future clinical research studies.

## 1 Introduction

Sodium ions (Na^+^) play a crucial role in cardiomyocyte excitability and thus in the contractile function of the human heart [1]. Accordingly, alterations of the myocardial tissue sodium concentration (TSC) can serve as indicators for pathological changes [2]. For instance, elevated myocardial TSC has been observed in patients with myocardial infarction [3] and primary hyperaldosteronism [4]. In this context, sodium (^23^Na) MRI enables noninvasive in vivo measurement of the myocardial TSC [1].

However, ^23^Na MRI faces significant challenges due to the lower MR sensitivity of ^23^Na compared to hydrogen (^1^H), resulting in a signal‐to‐noise ratio (SNR) approximately 6000 times lower in myocardial tissue [1]. To address this, larger voxel sizes, longer acquisition times, and ultrahigh field strengths such as 7 T are commonly used to improve SNR [5]. Due to the limited spatial resolution of ^23^Na MRI, additional high‐resolution ^1^H MR images are required for accurate myocardial and blood pool segmentation, enabling partial volume correction (PVC) [6], and reliable TSC quantification [7].

Conventionally, ^23^Na and ^1^H MRI are acquired separately, often using different pulse sequences and sometimes even at different field strengths, which increases scan time and introduces susceptibility to physiological changes or subject motion between scans [7]. To overcome these limitations, we employed a time‐efficient, dual‐nuclear interleaved ^23^Na/^1^H pTx MR sequence [8] enabling quasi‐simultaneous ^23^Na and ^1^H MR data acquisition, while ensuring homogenous ^1^H excitation via parallel transmission (pTx) [9].

In cardiac ^23^Na MRI, physiological motion presents additional challenges, as both breathing and cardiac motion can corrupt TSC quantification [7]. While methods such as respiratory self‐gating [7, 10] and external cardiac logging enable retrospective motion‐resolved reconstructions, they reduce acquisition efficiency by discarding large portions of the data. Motion correction using interleaved acquired ^23^Na and ^1^H acquisitions has previously been demonstrated for rigid head movements in ^23^Na brain MRI [11], but is not directly applicable to physiological cardiac and respiratory motion due to temporal resolution constraints and the nonrigid contraction of the heart. Thus, in this work, we implemented a new motion correction approach for interleaved cardiac ^23^Na/^1^H MRI, combining rigid respiratory and nonrigid cardiac motion correction based on high‐resolution ^1^H data.

Another factor influencing the ^23^Na quantification is the spatial variation of the B_1_ field generated by the radio frequency (RF) coil. The combined ^23^Na/^1^H torso RF coil employed in this work exhibits nonuniform ^23^Na transmit (B_1_^+^) and receive (B_1_^−^) field profiles [8], which are additionally influenced by body shape and subject positioning. While B_1_^+^ can be measured in vivo [12], B_1_^−^ must typically be estimated from phantom data [10] or electromagnetic RF field simulations [7], potentially introducing errors. To reduce these effects, we developed a novel transmit and receive (B_1_) bias field correction for cardiac ^23^Na MRI. It utilizes a synthetic ^23^Na prior image, derived from ^1^H segmentations of the myocardium and blood, to estimate and correct for low‐frequency B_1_ related signal bias.

The proposed correction methods were first validated in ^23^Na/^1^H simulations incorporating realistic respiratory and cardiac motion [13], as well as B_1_ bias field effects. To assess the impact of motion and B_1_ corrections on the repeatability of myocardial TSC quantification in vivo, a repeatability study was performed with 10 healthy subjects.

## 2 Methods

### 2.1 Simulations

**FIGURE 1:**
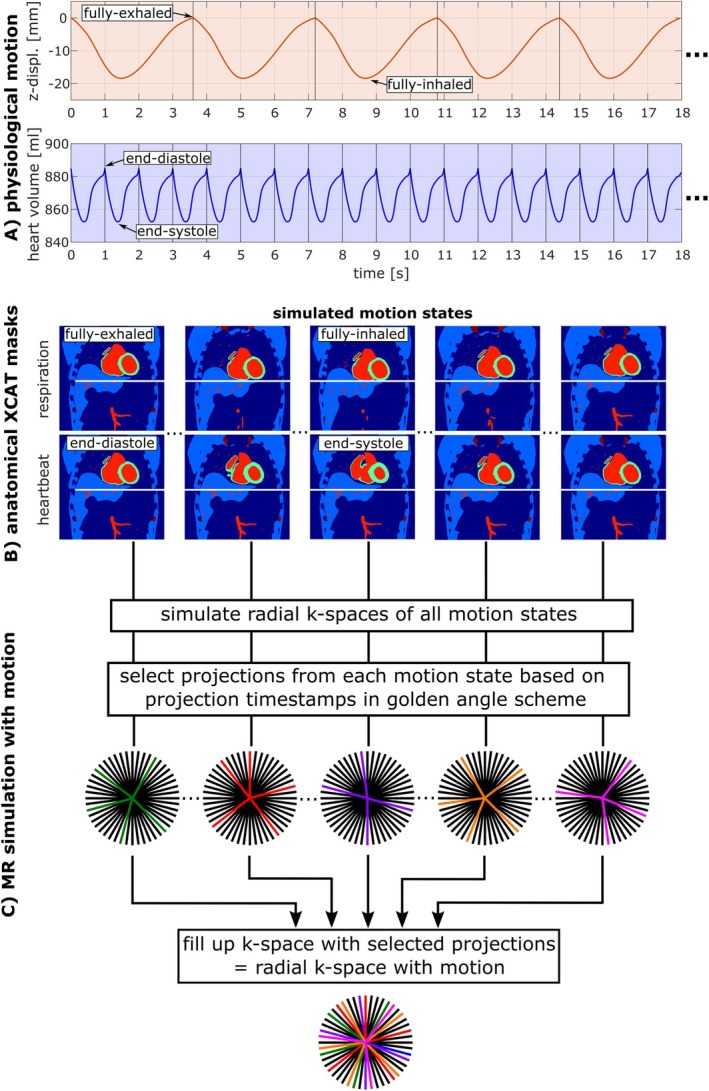
Simulation scheme: XCAT simulation with realistic respiration and heartbeat, sampled at a temporal resolution of 60 ms. A respiratory cycle of 3.6 s was chosen, while the heartbeat was simulated with a cycle duration of 1 s (A). The motion states for respiration and heartbeat were simulated independently. The resulting anatomical XCAT masks (B, only representative states shown here) of all distinct motion states were then used as inputs for ^23^Na and ^1^H MR simulations (C). Specific projections of each simulated k‐space were selected—based on their temporal alignment with the respiratory and cardiac cycle—and subsequently combined into a single composite k‐space.

The 4D extended cardiac‐torso (XCAT) simulation phantom [14] is a computational model that simulates realistic anatomical structures and physiological motions based on real human imaging data. It enables the generation of anatomical compartment masks (1 mm isotropic spatial resolution) of the human torso, in particular the heart, during different timepoints of the respiratory and cardiac cycle (Figure 1B). In this study, respiratory and cardiac motion were simulated separately: the heart remained in the end‐diastolic phase during respiratory motion simulation, and in the fully‐exhaled state during cardiac motion simulation. For the respiratory simulation, the maximal heart motion range was set to 18.4 mm in SI, 2.4 mm in AP, and 0 mm in LR direction [15], with a cycle duration of 3.6 s. The cardiac simulation used a 1 s cycle (heart rate of 60 bpm, systole: 400 ms, diastole: 600 ms) (see Figure 1A). Both simulations were sampled at a temporal resolution of 60 ms, yielding 50 distinct cardiac and 60 distinct respiratory motion states, which are repeated periodically.

For each motion state, individual ^23^Na and ^1^H MR simulations of the radial k‐space data were performed using binary masks from the XCAT phantom. The MR simulations [16] incorporated ^23^Na and ^1^H specific tissue parameters (see Tables S1 and S2) as well as sequence parameters similar to the used interleaved sequence (Section 2.2). To simulate the influence of the physiological motion, ^23^Na and ^1^H projections were selected from the MR simulations of the different respiratory/cardiac motion states—based on the acquisition timestamps within the sequence scheme—and assembled into a composite radial k‐space for each nucleus (Figure 1C).

### 2.2 Measurements

#### 2.2.1 Measurement Setup

Measurements were performed at a 7 T whole‐body MR system (MAGNETOM Terra.X, Siemens Healthineers, Erlangen, Germany) using a combined ^23^Na/^1^H RF coil setup (Rapid Biomedical, Rimpar, Germany) [8]. The setup consists of a ^23^Na RF volume coil and two 4Tx/8Rx ^1^H torso arrays allowing for interleaved ^23^Na/^1^H acquisitions and the use of pTx for ^1^H MRI.

In order to assess the repeatability of the myocardial TSC quantification, 10 healthy subjects (3 female, 27.1±3.5 years, BMI: $24.2\pm 2.3\;\mathrm{kg}/m^{2}$) were examined twice using the described MR imaging protocol consisting of the interleaved ^23^Na/^1^H pTx sequence and ^23^Na B_1_^+^ mapping. After the first measurement block, the subjects stood up and were then repositioned within the coil. Venous blood samples were collected from each subject before the measurement and the Na^+^ concentration in the blood serum as well as hematocrit was determined in the laboratory. All measurements were approved by the local ethics committee and written informed consent was obtained from all subjects prior to the examination.

#### 2.2.2 Interleaved ^23^Na/ ^1^H pTx Sequence

**FIGURE 2:**
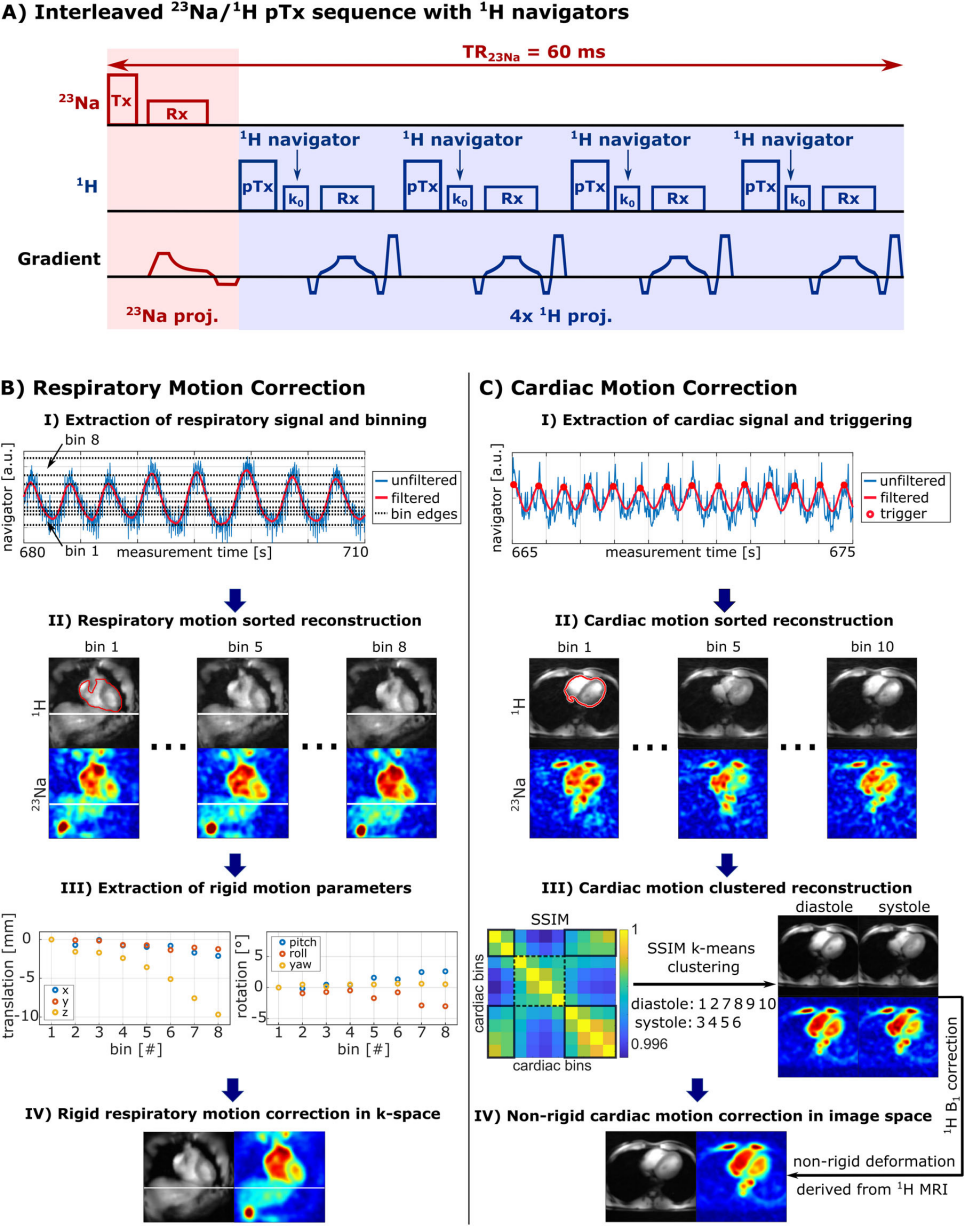
Respiratory and cardiac motion correction algorithm for interleaved acquired ^23^Na/^1^H MRI. For the extraction of the respiratory and cardiac signal, additional ^1^H navigators (called k_0_) were added to the interleaved acquisition scheme (A). For the respiratory motion correction (B), the ^1^H navigators are utilized to extract a respiratory signal (I). Based on the filtered respiratory signal, the acquired ^23^Na and ^1^H data are assigned into eight different respiratory bins allowing to perform a motion‐sorted reconstruction (II). The motion‐sorted ^1^H images are then rigidly co‐registered to the exhaled image (here: Bin 1) within the heart region (red outline), resulting in three translational and three rotational transformation parameters for each bin (III). Finally, these parameters are used to correct the ^1^H as well as the ^23^Na data directly in the k‐space (IV, reconstructed images shown). The cardiac motion correction (C) is performed after applying the respiratory motion correction on the ^23^Na and ^1^H data. Similar to the respiratory motion correction, the cardiac motion signal is retrieved from the ^1^H navigators (I). Based on the maxima of the cardiac motion signal, the ^23^Na and ^1^H projections are sorted into ten different cardiac phases allowing to reconstruct cardiac motion‐sorted images (II). The ten cardiac phases are clustered into a diastolic and systolic phase based on the ^1^H SSIM within the heart region (III). Then the diastolic and systolic ^1^H images were B_1_ bias corrected and the systolic ^1^H image was nonrigidly co‐registered to the diastolic image. Subsequently, the same deformation field was applied to the systolic ^23^Na image. Adding up the diastolic and co‐registered systolic ^23^Na and ^1^H images results in cardiac motion‐corrected ^23^Na and ^1^H images (IV).

An interleaved ^23^Na/^1^H pTx sequence [8] with a density‐adapted 3D radial [17] readout was utilized to acquire both ^23^Na and ^1^H MR data within one measurement: (∆*x*_23Na_)^3^ = (6 mm)^3^, (∆*x*_1H_)^3^ = (2 mm)^3^, FA_23Na_ = 82°, FA_1H_ = 10°, TR_23Na_ = 60 ms, TE_23Na_ = 1.15 ms, TA = 15 min. A full overview of all sequence parameters is provided in ref. [8]. For ^1^H excitation, a universal cardiac phase shim [18] was employed. As the data were acquired during free breathing, navigators were added to the sequence to enable retrospective respiratory and cardiac self‐gating [10] (Figure 2A). The navigators consisted of 10 additional data points of the ^1^H k‐space center signal, called k_0_, which were acquired before each ^1^H projection. The radial projections of both nuclei were acquired in a 3D golden angle scheme [19] to allow motion‐sorted reconstruction of the data. For validation purposes, an additional electrocardiogram was recorded simultaneously to the interleaved sequence for 3 of the 10 subjects.

All data were reconstructed on a standalone PC (Intel Core i7‐9700K CPU 8 cores @ 3.60 GHz with 128 GB RAM) using a custom‐written MATLAB (The Math‐Works, Natick, MA, USA; version 2020b) script, including the application of a Hamming filter, a nonuniform fast Fourier transform [20] as well as a correction for gradient field nonlinearities [21]. ^23^Na images were zero‐filled to 2 mm isotropic resolution to match the ^1^H MRI. For analyses with myocardium and blood masks, simulated ^23^Na images were zero‐filled to 1 mm isotropic to match the XCAT mask resolution.

### 2.3 Motion Gating and Corrections

To extract respiratory and cardiac motion during the interleaved ^23^Na/^1^H acquisition, the k_0_ navigator of the ^1^H signal was used. Ten central k‐space samples ($T_{\text{dwell}}=7.6\;\mathrm{\mu s}$) per ^1^H projection were averaged, and gradient timing mismatches were corrected [22]. As ^1^H data were acquired with alternating TRs (13.08 and 20.76 ms), the navigator signal was interpolated to a uniform 15 ms resolution for easier processing.

#### 2.3.1 Respiratory Motion Gating and Correction

Respiratory motion was estimated by filtering the k_0_ navigator signals of all 16 ^1^H receive channels using a moving average filter with a 1.5 s window. This suppresses cardiac‐related signal fluctuations, as their cycle duration typically lies below this threshold (heart rates > 40 bpm). Based on the navigator signal of each channel, images corresponding to the fully‐inhaled and fully‐exhaled states, that is, bins 1 and 8, were reconstructed and rigidly co‐registered within the heart region using Elastix [23]. The channel leading to the maximum displacement in the superior–inferior (SI) direction between these two respiratory phases was selected as the most sensitive to respiratory motion and was used as the final respiratory navigator channel (Figure 2A—I).

The extracted navigator signal was used to bin both ^1^H and ^23^Na projections into $N_{\text{resp}}$ respiratory states, each containing an equal number of projections. These bins were reconstructed to generate $N_{\text{resp}}$ respiratory‐gated images (Figure 2A—II). All gated ^1^H images were then rigidly co‐registered to the fully‐exhaled state, and the resulting transformation parameters (three translations, three rotations, Figure 2A—III) were applied to the corresponding k‐space subsets of both ^1^H and ^23^Na data [11, 24], yielding respiratory motion–corrected images reconstructed in the exhaled state (Figure 2A—IV).

#### 2.3.2 Cardiac Motion Gating and Correction

Cardiac motion correction was performed after respiratory motion correction. For each ^1^H receive channel, the frequency power spectrum of the k_0_ navigator signal was computed and denoised using a Savitzky–Golay filter [25] (*sgolayfilt* in MATLAB's Signal Processing Toolbox). Within the expected cardiac frequency range (0.5–2 Hz), the dominant peak was identified using MATLAB's *findpeaks* function (Signal Processing Toolbox), and its prominence ($p_{\mathrm{card}}$), center frequency ($f_{\mathrm{card}}$), and width ($w_{\mathrm{card}}$) were extracted. The total power of the remaining spectrum ($S_{\text{noise}}$) was calculated, and the channel with the highest prominence‐to‐noise ratio ($p_{\mathrm{card}}/S_{\text{noise}}$) was selected.

This channel's navigator signal was bandpass‐filtered using a subject‐specific Butterworth filter [26] with cutoffs $f_{\mathrm{cut}}=f_{\mathrm{card}}\pm 2\cdot w_{\mathrm{card}}$. The resulting peaks served as cardiac triggers (Figure 2B—I), which were validated against ECG signals in three subjects (Figure S1).

^1^H and ^23^Na projections were sorted into 10 cardiac phases based on the time since the last trigger. Structural similarity indices [27] (SSIM) of cardiac‐gated ^1^H images (Figure 2B—II) were used for k‐means clustering [28, 29] into systolic and diastolic phases. The clustered projections were reconstructed into diastolic and systolic gated ^1^H and ^23^Na images (Figure 2B—III). Subsequently, nonrigid registration between the B_1_ bias corrected (see Section 2.7) systolic and diastolic ^1^H images was performed using Elastix [23] to estimate cardiac motion fields from the ^1^H images. These motion fields were then applied to the systolic ^23^Na and ^1^H images. Finally, averaging of the diastolic and motion‐corrected systolic images yielded cardiac motion–corrected ^23^Na and ^1^H images in the diastole (Figure 2B—IV).

Respiratory motion‐sorted reconstruction into 8 bins required ∼1.5 h, with motion parameters estimated in 3 min. Cardiac motion‐sorted reconstruction into 10 and 2 bins took ∼2 h and ∼1 h. Cardiac motion correction was executed in ∼3 min.

### 2.4 Segmentation

For the in vivo data, initial manual segmentations of the left myocardium and blood pool were performed on the interleaved acquired ^1^H images with B_1_ bias correction (see Section 2.7) but without any motion corrections. These 20 manual segmentations (10 subjects, each measured twice) were then used to train a 3D nnUNet [30] with both ^23^Na and ^1^H images as inputs, enabling automatic prediction of myocardium and blood pool for motion‐corrected ^23^Na and ^1^H images. The predicted masks of the blood pool were truncated above the myocardial voxel with the highest spatial position in the superior–inferior direction to exclude the great vessels in the evaluation.

### 2.5 Partial Volume and Relaxation Correction

To improve the accuracy of the ^23^Na quantification, a PVC [6] was applied. Point‐spread functions (PSFs), including *T*_2_* values from literature (Table S1), were convolved with myocardial and blood masks to obtain region‐spread functions (RSFs). These were used to correct for the ^23^Na signal spillover between compartments.

Additionally, relaxation effects on the ^23^Na signals were corrected using the formula

(1) $$S_{\text{corr}}\left(\vec{x}\right)=\frac{S_{\text{uncorr}}\left(\vec{x}\right)}{\;r\cdot \exp\left(-\frac{\mathrm{TE}}{T_{2,s}^{*}}\;\right)+\left(1-r\right)\cdot \exp\left(-\frac{\mathrm{TE}}{T_{2,l}^{*}}\right)}\cdot \frac{1-\cos\left(\mathrm{FA}\right)\exp\left(-\frac{\mathrm{TR}}{T_{1}}\right)}{1-\exp\left(-\frac{\mathrm{TR}}{T_{1}}\right)}$$

and the corresponding relaxation parameters for myocardium and blood (see Table S1). The flip angle (FA) used in this formula was the subject‐specific median FA within the blood pool, measured with the actual flip‐angle imaging (AFI) sequence (see Section 2.7).

### 2.6 TSC Quantification

The sodium concentration $c_{Na^{+},\text{blood}}$ in whole blood was calculated using the blood serum sodium concentration $c_{{\mathrm{Na}}^{+},\;\text{serum}}$ and hematocrit values obtained from laboratory analysis:

(2) $$c_{{\mathrm{Na}}^{+},\;\text{blood}}=\left(1-\text{hematocrit}\right)\cdot c_{{\mathrm{Na}}^{+},\;\text{serum}}$$

Next, the ventricular blood pool served as an internal reference for ^23^Na MRI to derive quantitative myocardial apparent tissue sodium concentrations (aTSC) by linear calibration:

(3) $$\mathrm{aTS}C_{\mathrm{myo}}=c_{{\mathrm{Na}}^{+},\;\text{blood}}\cdot \frac{{\bar{S}}_{\mathrm{myo}}}{{\bar{S}}_{\text{blood}}}$$

The term “apparent” accounts for potentially remaining relaxation weighting, as suggested by Stobbe and Beaulieu [31].

### 2.7 B_1_ Correction

To correct for ^23^Na signal variations caused by the spatially varying B_1_^+^ and B_1_^−^ field distributions of the employed RF coil, we developed a novel B_1_ bias field correction method. Previous approaches assumed a homogeneous signal distribution across the entire organ [32, 33], which is sufficient for qualitative ^1^H images but inadequate for quantitative ^23^Na MRI due to differing sodium concentrations in the blood pool and myocardium. In order to address this, we incorporated RSFs of the blood pool and myocardium into our B_1_ bias correction algorithm:

1. RSFs of myocardium and blood were computed from ^1^H segmentations, assuming homogeneous signal and accounting for partial volume blurring.
2. A synthetic prior image was generated by adding the RSFs with an assumed myo‐blood ratio $r_{\text{assumed}}$.
3. The prior and original ^23^Na images were masked to the heart and low‐pass filtered using *imgaussfilt3* (Image Processing Toolbox) with standard deviation $\sigma_{23\mathrm{Na}}$ = 10 mm (optimization in Figure S2).
4. The B_1_ bias field was estimated as
(4) $$B_{1,\text{bias}}\left(\vec{x}\right)=\frac{S_{\text{orig},\text{filt}}\left(\vec{x}\right)}{S_{\text{prior},\text{filt}}\left(\vec{x}\right)}\approx \sin\left(\mathrm{FA}\left(\vec{x}\right)\right)\cdot B_{1}^{-}\left(\vec{x}\right)$$
  yielding an approximation of the low‐frequency bias, arising from the combined effects of B_1_^+^ and B_1_^−^ inhomogeneities.
5. The B_1_ bias correction was applied voxel‐wise in the heart:
(5) $$S_{B1,\text{corr}}\left(\vec{x}\right)=\frac{S_{\text{uncorr}\left(\vec{x}\right)}}{B_{1,\text{bias}}\left(\vec{x}\right)}$$
6. PVC was then applied to the mean myocardium and blood ^23^Na signals of the B_1_ corrected image to obtain the measured myo‐blood ratio $r_{\text{measured}}$.

Because the true ratio is unknown, steps (2–6) were iterated for $r_{\text{assumed}}$∈ [0.3, 0.7] (step size 0.005), and the value yielding the smallest difference $\left|r_{\text{measured}}-r_{\text{assumed}}\right|$ was selected. For the ^1^H B_1_ correction, only steps (2–5) were used, with a homogeneous support region instead of a synthetic prior and a Gaussian kernel with standard deviation $\sigma_{1H}$ = 12 mm. A detailed overview of the implemented B_1_ bias corrections can be found in Figure S3. The iterative ^23^Na B_1_ bias correction of the in vivo measurements took around 5 min per subject and the ^1^H B_1_ bias correction below 1 s.

In addition, fast B_1_^+^ mapping for the ^23^Na RF coil was performed using the AFI [34] sequence ((Δ *x*)^3^ = (12 mm)^3^, FA = 60°, TR_1_/TR_2_ = 12/48 ms, TE = 1.05 ms, TA = 3 min, randomized *xy*‐spoiling [35]). The resulting FA maps were smoothed using a Gaussian kernel ($\sigma_{\mathrm{AFI}}$ = 14 mm) and employed to estimate the mean absolute FA within the blood pool for relaxation correction (Equation 1). The commonly used double angle method requires a long TR (∼250 ms) and two different flip angles (45° and 90°), reducing acquisition efficiency and potentially introducing errors from tissue‐specific relaxation [36]. This is not the case for the AFI sequence using only one pulse for excitation as well as shorter TRs (12 and 48 ms), which enables more efficient k‐space sampling (Figure S4). Accelerating the AFI acquisition from 15 to 3 min had minimal impact on the FA maps (Figure S4).

The proposed B_1_ bias field correction method was compared to two other commonly used methods: (1) B_1_^−^ and B_1_^+^ maps derived from phantom measurements [8] and (2) phantom‐derived B_1_^−^ map combined with in vivo B_1_^+^ maps obtained from the AFI measurements.

### 2.8 Evaluation Procedures

^23^Na and ^1^H simulations were performed 20 times with different spatially distributed noise approximating the in vivo SNR. For simulated ^23^Na MRI, the voxel‐wise SNR was computed from the mean and standard deviation across all 20 simulations [37]. The blood SNR was obtained by averaging over the entire blood pool, and myo–blood signal ratios were computed by dividing the mean myocardium ^23^Na signal by the mean ^23^Na blood signal, calculated using masks from the corresponding ground truth state. Additionally, the normalized root mean square error (NRMSE) relative to the ground truths was calculated within the heart. The ground truths were defined as simulated images representing the fully‐exhaled state for respiratory motion, the end‐diastolic state for cardiac motion, and a B_1_ bias–free image for B_1_ correction.

To evaluate the ^23^Na B_1_ bias correction across different myocardial TSC values, simulations were performed for TSCs between 30.1 and 55.9 mM (blood fixed at 81 mM), each repeated 20 times with different noise distributions. A B_1_ bias field was generated from phantom‐measured B_1_^+^/B_1_^−^ maps [8] using $B_{1,\text{bias}}\left(\vec{x}\right)\approx \sin\left(\mathrm{FA}\left(\vec{x}\right)\right)\cdot B_{1}^{-}\left(\vec{x}\right)$ and applied to the GT images to create biased images. Three heart positions were simulated by shifting the bias field relative to the images. The ^23^Na B_1_ bias correction was then applied, and corrected and uncorrected images were compared to the GT using NRMSE and myo‐blood ratios.

For in vivo ^23^Na MRI, SNR values were estimated for myocardium and blood using background noise region‐of‐interests [37]:

(6) $$\mathrm{SN}R_{\mathrm{myo}/\text{blood}}=\frac{{\text{mean}}_{\mathrm{myo}/\text{blood}}}{{\mathrm{std}}_{\text{noise}}}\cdot \sqrt{\frac{4-\pi }{2}}$$

This analysis was performed only for rigid respiratory motion correction, as it is unsuitable for nonrigid cardiac motion correction, which alters noise properties.

To evaluate the repeatability of the in vivo myocardial aTSC quantification, the coefficient of repeatability (CR) was calculated using the formula

(7) $$\mathrm{CR}=2.77\cdot \sqrt{\frac{1}{10}\sum_{i=1}^{10}\sum_{k=1}^{2}{\left(\mathrm{aTS}C_{i,k}-{\bar{\text{aTSC}}}_{i}\right)}^{2}}$$

with ${\bar{\text{aTSC}}}_{i}=\frac{\mathrm{aTS}C_{i,1}+\mathrm{aTS}C_{i,2}}{2}$ as described in ref. [38]. The relative CR (rCR) was obtained by dividing the CR by the global mean aTSC value. Additionally, the results were visualized using Bland–Altman plots [39].

## 3 Results

### 3.1 Validation of Respiratory and Cardiac Motion Correction in Simulation Study

**FIGURE 3:**
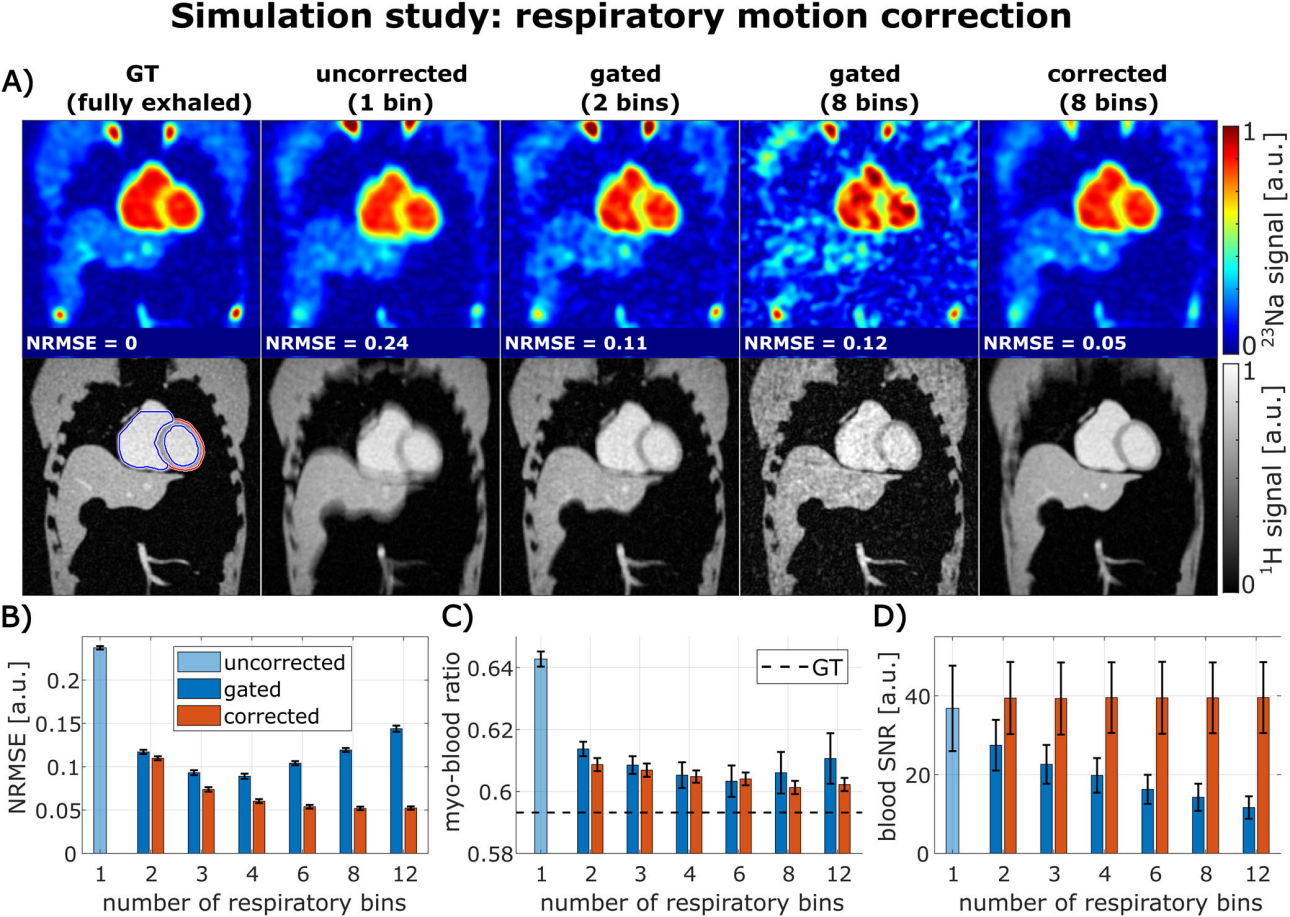
Validation of the respiratory motion correction in simulation study (respiratory motion: 18.1/2.4/0 mm in SI/AP/LR directions; no cardiac motion: End‐diastolic motion state used). Exemplary ^23^Na and ^1^H images are shown in (A). The use of respiratory gating to reduce respiratory motion effects (B, C) results in a decrease in SNR (D). Motion correction shows improved compensation of the respiratory motion compared to respiratory gating (lower NRMSE and myo‐blood ratio closer to GT), while showing no decrease in SNR (D). Performing the respiratory motion correction with eight respiratory bins resulted in the lowest NRMSE (B) and a myo‐blood ratio closest to the GT (C).

The respiratory motion correction was validated using ^23^Na and ^1^H simulations containing realistic respiratory motion. Representative reconstructed images are shown in Figure 3A. The ^1^H images demonstrate that gating data into more respiratory bins (e.g., 8 instead of 2)—resulting in fewer projections per bin—leads to reduced respiratory motion blurring compared to the uncorrected image. However, this also increases undersampling artifacts and reduces SNR.

In contrast, the proposed respiratory motion correction further decreases quantification errors in ^23^Na MRI, achieving lower NRMSE values (B) and myo‐blood ratios (C) closer to the GT (fully‐exhaled motion state) than respiratory gating, while maintaining the original SNR (D). Based on the NRMSE analysis, using 8 respiratory bins for motion correction resulted in the lowest NRMSE (B), indicating the best quantitative agreement to the GT. Consequently, eight respiratory bins were used for the respiratory motion correction of the in vivo data.

**FIGURE 4:**
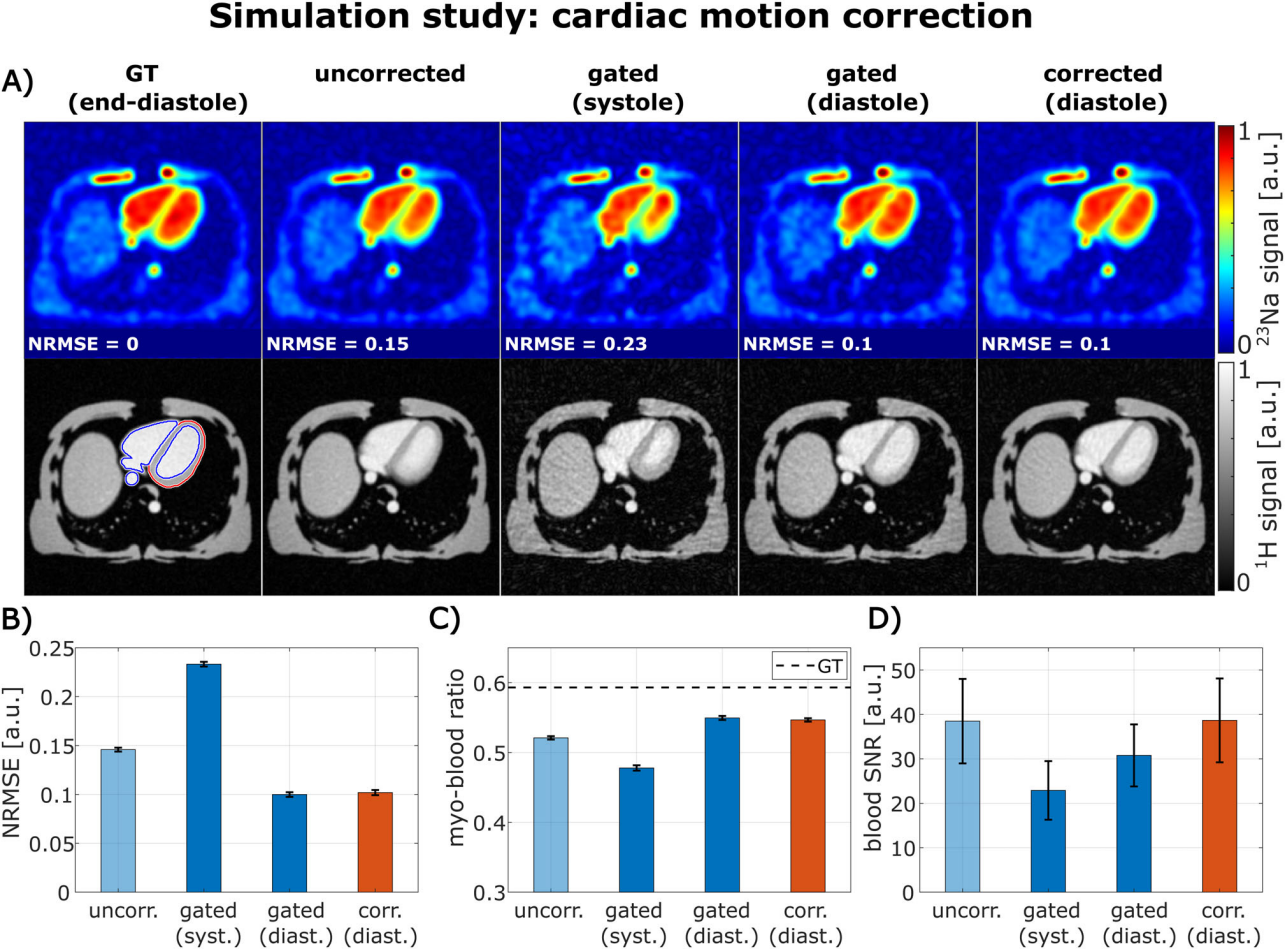
Validation of the cardiac motion correction in simulation study (no respiratory motion: Fully‐exhaled motion state used; cardiac motion: 60 bpm, systole: 0–400 ms, diastole: 400–1000 ms). Exemplary ^23^Na and ^1^H images are shown in (A). Cardiac diastolic gating and motion correction into the diastolic phase reduces the NRMSE (B) as well as the deviation of the myo‐blood ratio to the GT (C) compared to the uncorrected image. Cardiac motion correction enables this while providing a higher SNR than cardiac gating (D).

Cardiac motion correction was validated in a simulation study with realistic cardiac motion (Figure 4). Both diastolic‐gated as well as motion‐corrected images (A) showed a reduction in NRMSE (B) compared to the uncorrected image and ^23^Na myo‐blood signal ratios (C) closer to the GT, demonstrating decreased quantification errors caused by cardiac motion. In addition, applying the cardiac motion correction increased the SNR by 25% compared to the diastolic‐gated images (D).

### 3.2 Validation of B_1_ Bias Field Correction in Simulation Study

**FIGURE 5:**
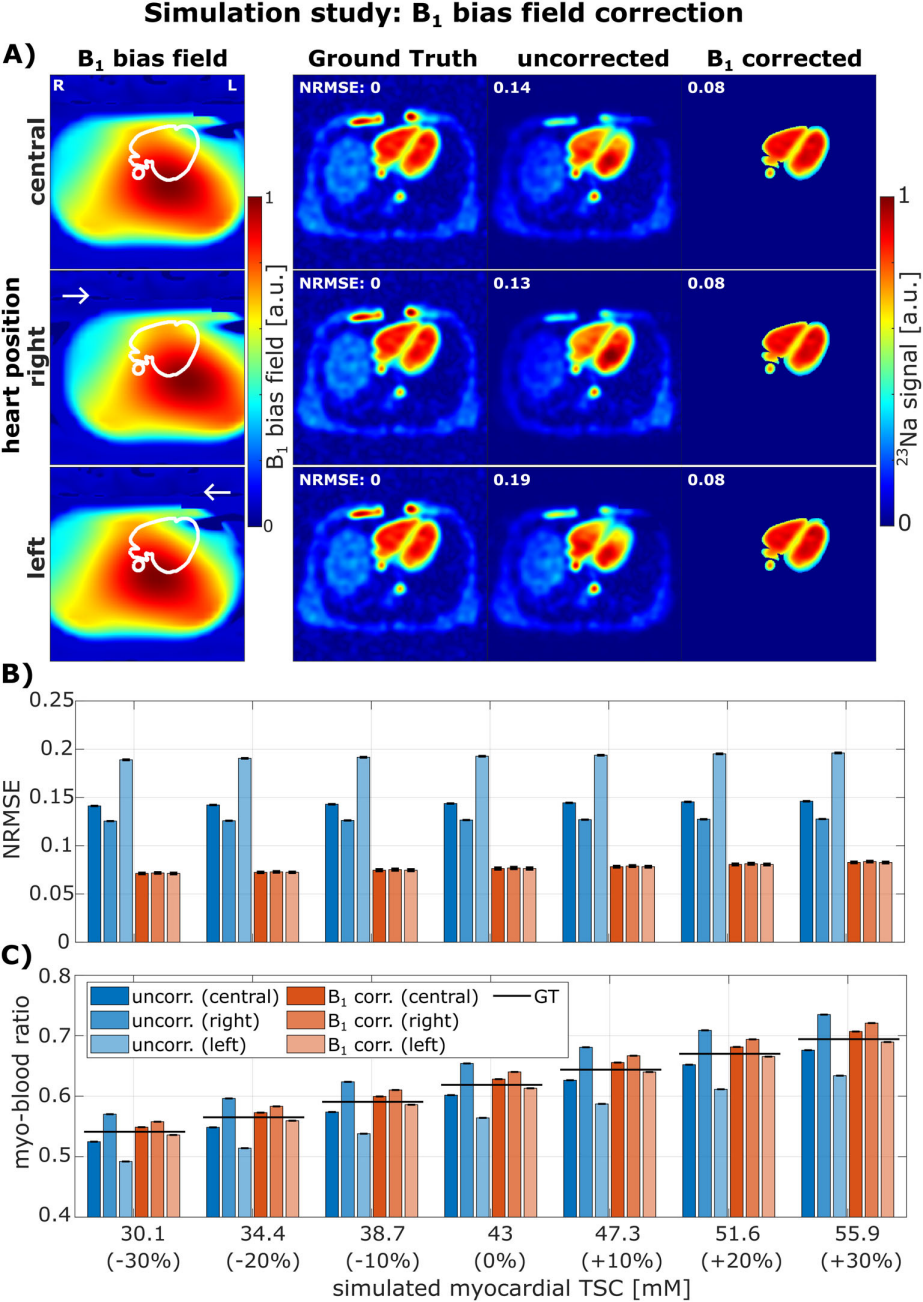
Validation of the B_1_ bias field correction in simulation study (no respiratory motion: Fully‐exhaled motion state used; no cardiac motion: End‐diastolic motion state used). Note that since the B_1_ bias field correction was applied only within the heart, the B_1_ corrected images were masked accordingly. After applying B_1_ bias correction, the ^23^Na signal distribution appeared more homogeneous within the heart (A, images shown for 43 mM myocardial aTSC). Across all simulated TSC values and all three positions, the B_1_ correction consistently reduced NRMSE over the heart (B). Additionally, for every position and TSC value, the myo‐blood ratios (masks see Figure 4) after B_1_ correction were closer to the GT than those obtained without correction (C).

The B_1_ bias field correction was validated in a simulation study incorporating three different B_1_ bias field distributions within the heart (Figure 5). After applying the proposed B_1_ bias correction, the ^23^Na signal appeared more homogeneous across the heart (A). NRMSE values (B) within the heart region as well as myocardium‐blood ratios of the ^23^Na signal (C) confirmed that the B_1_ bias correction effectively reduced quantification errors for all three heart positions and all simulated myocardial TSC values (30.1–55.9 mM). The ^23^Na B_1_ bias correction also demonstrated robust performance in simulations with myocardial T*_2,short_ deviating from the assumed 3 ms (Figure S5—I) as well as in simulations with hypertrophied myocardium (Figure S6B—II).

### 3.3 In Vivo Application of Respiratory and Cardiac Motion Correction

**FIGURE 6:**
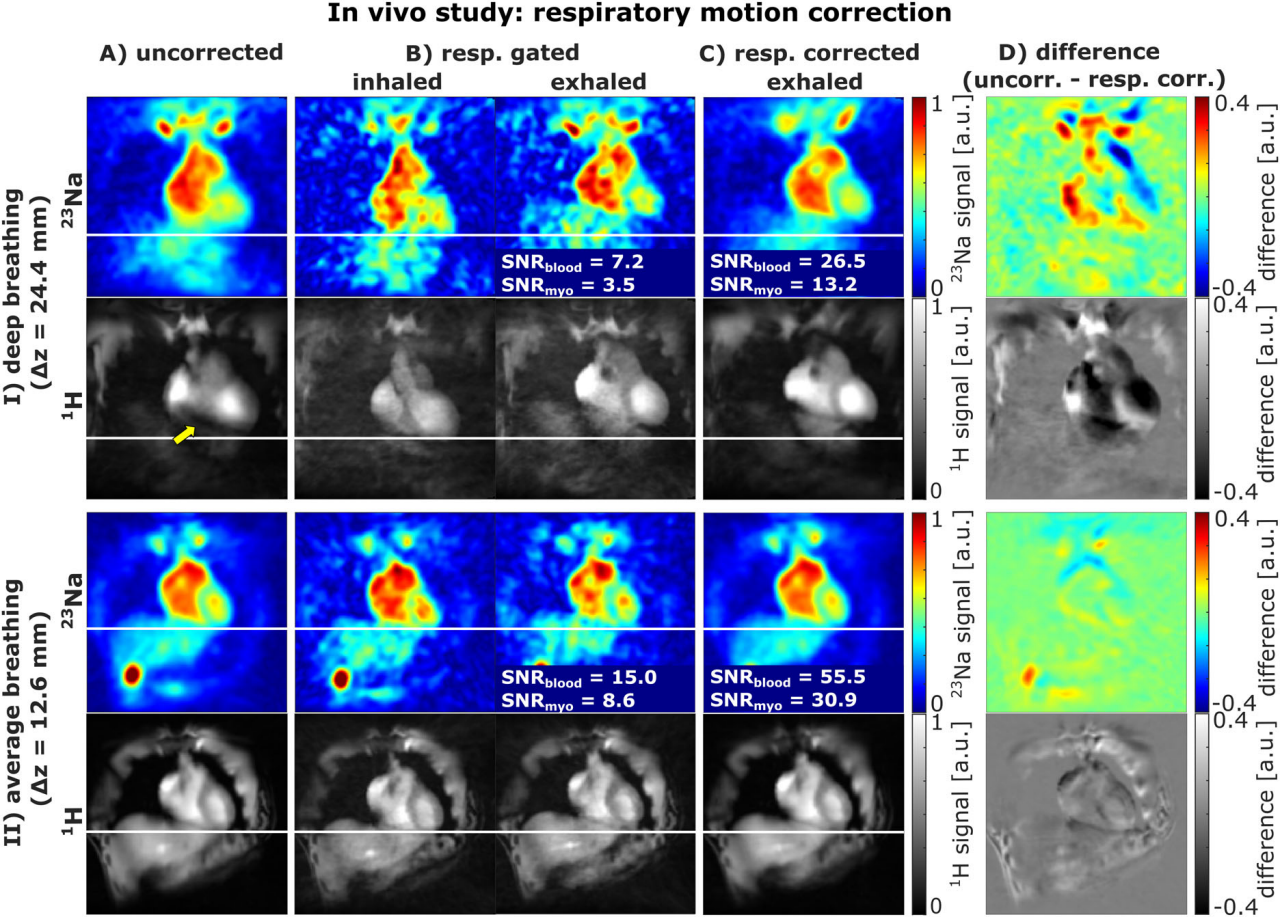
Influence of respiratory motion gating and correction on ^23^Na and ^1^H images for the in vivo measurements of two subjects with different breathing depth. For the subject with deep breathing (I), the uncorrected image (A) exhibits severe motion blurring and even signal dropouts in the ^1^H MRI (yellow arrow). With a more average breathing depth (II), these artifacts are less pronounced. Respiratory‐gated reconstruction (B) reduces motion‐induced blurring in both subjects and visualizes the respiratory displacement between the fully‐inhaled and fully‐exhaled bins. Applying respiratory motion correction (C) diminishes motion‐related errors while preserving higher SNR than respiratory gating. The quantitative impact of the respiratory motion correction is evident in the difference maps (D) and is particularly pronounced for the subject with deep breathing (I).

Figure 6 illustrates the impact of respiratory motion correction on in vivo ^23^Na and ^1^H MRI for two subjects with different breathing depths. In the deep‐breathing subject (I; 24.4 mm displacement), uncorrected images (A) show strong blurring and ^1^H signal dropouts, whereas these effects are less pronounced for the subject with average breathing (II). Respiratory gating into eight bins (B) reduces blurring but uses only one‐eighth of the data, lowering SNR. In contrast, respiratory motion correction (C) aligns all projections to the exhaled state, improving sharpness while preserving SNR. Across all 20 measurements, motion correction increased SNR by factors of 3.56±0.37 in the myocardium and 3.57±0.36 in the blood pool compared to gated exhaled state images. Difference maps (D) highlight the stronger effect for the deep‐breathing subject.

**FIGURE 7:**
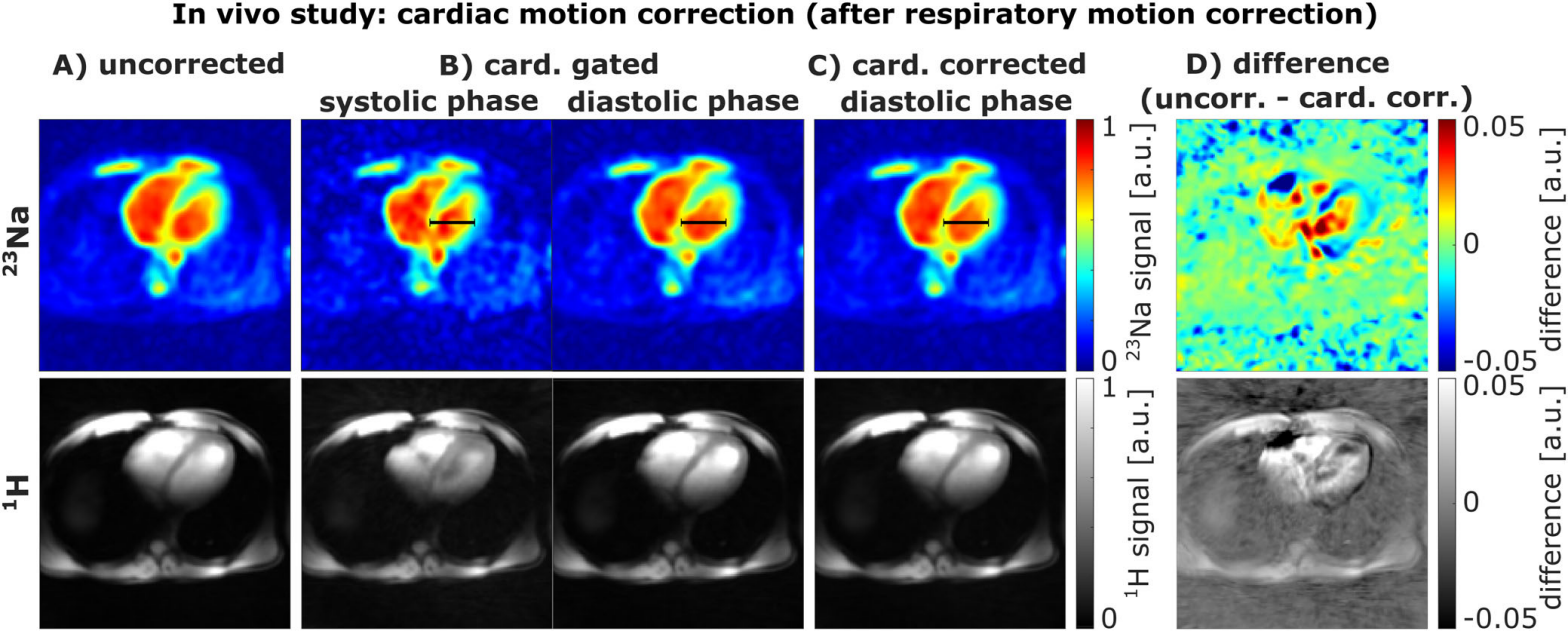
Influence of cardiac motion gating and correction on ^23^Na and ^1^H images for one in vivo measurement. Note that the cardiac gating and motion correction is applied after respiratory motion correction in the fully‐exhaled state. The correct assignment of the ^23^Na and ^1^H data into a systolic and diastolic phase is demonstrated by the increased width (black bar for reference) of the left ventricle in the diastolic compared to the systolic phase (B). The cardiac motion‐corrected image (C) displays good accordance to the cardiac‐gated diastolic image. The difference between the cardiac motion‐corrected and uncorrected image (A) shows quantitative changes in the region of the myocardium and blood pool (D).

Figure 7 illustrates the influence of cardiac motion on the ^23^Na and ^1^H images. Note that respiratory motion correction into the fully‐exhaled state has already been applied to the ^23^Na and ^1^H k‐space data prior to addressing cardiac motion. Compared to electrocardiograms recorded during measurements of three subjects, the cardiac self‐gating signal derived from the interleaved ^1^H data showed comparable trigger consistency (Figure S1).

Cardiac gating divided the data into 10 distinct phases, which were then clustered to reconstruct images corresponding to systolic and diastolic cardiac phases (B). The correct assignment of these phases is evident from the dilation of the left ventricle, appearing contracted during systole compared to diastole. Applying a nonrigid motion correction to the systolic image results in a cardiac motion‐corrected image in the diastolic phase (C). Compared to the uncorrected ^23^Na image (A), signal differences are visible in the myocardium and blood of the left ventricle (D).

### 3.4 In Vivo Application of B_1_ Bias Field Correction

**FIGURE 8:**
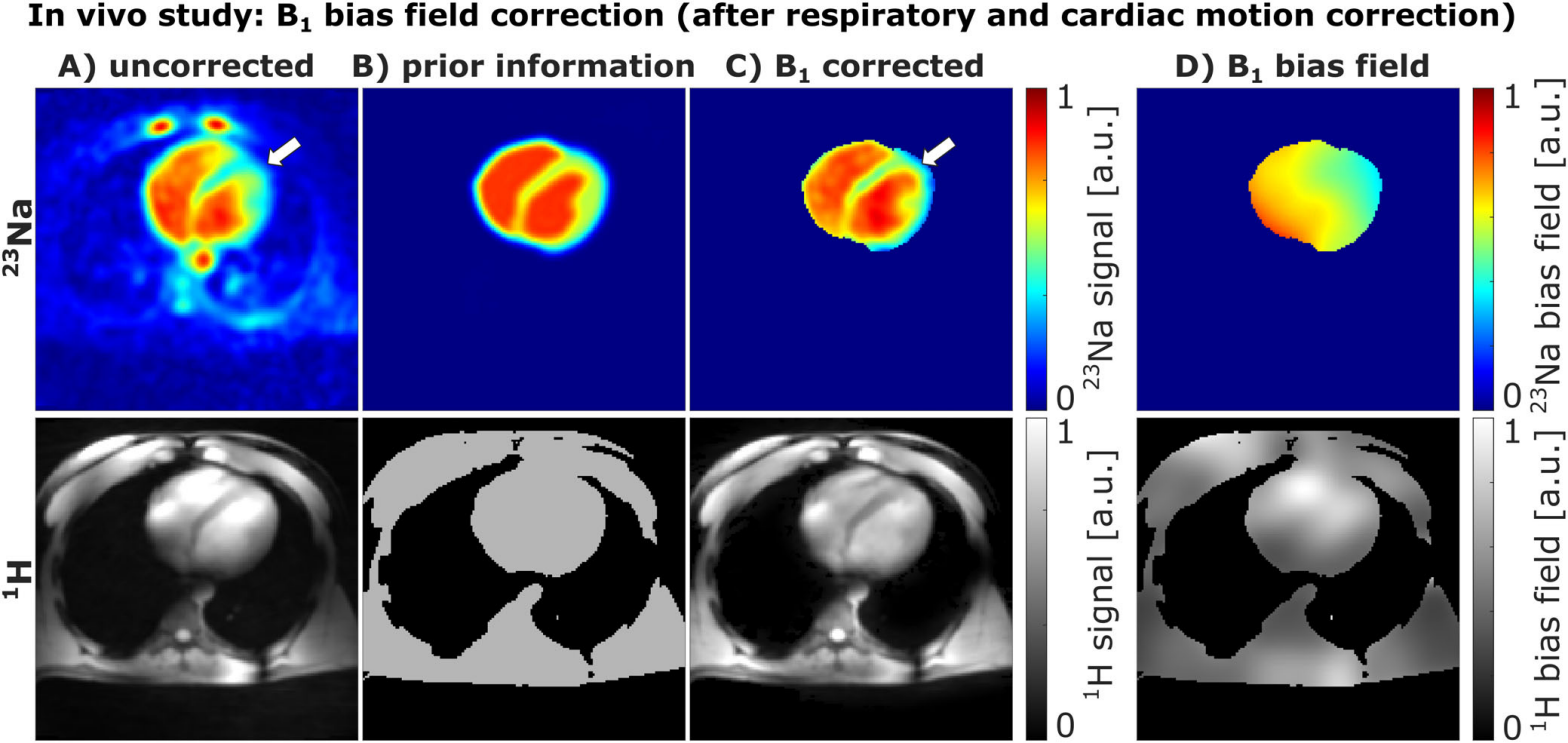
Influence of B_1_ bias field correction on ^23^Na and ^1^H images for one subject. (A) shows the respiratory and cardiac motion‐corrected ^23^Na and ^1^H image before B_1_ bias correction. For ^1^H MRI, a simple homogeneous support region (B) was used as anatomical prior to extract the ^1^H B_1_ bias field (D). After correction (C), the ^1^H image exhibits a more homogeneous signal distribution within the heart. For ^23^Na MRI, the prior information was generated based on ^1^H segmentations of the myocardium and blood (B). The estimated ^23^Na B_1_ bias field contained no anatomical structures (D). The B_1_ correction compensates for the signal loss on the top left of the heart (white arrow), leading to a more homogeneous signal distribution over the heart (C). Note that since the ^23^Na B_1_ bias field correction was applied only within the heart, the B_1_ corrected ^23^Na image (C) and the ^23^Na bias field (D) were masked accordingly.

Figure 8 shows the results of the B_1_ bias field corrections, applied to in vivo ^23^Na and ^1^H MRI of one subject. For ^1^H MRI, a homogeneous mask was created by thresholding and used as prior information (B). This allows obtaining a rough estimate of the ^1^H bias field (D), although some anatomical structures remain visible. Nonetheless, the B_1_ corrected ^1^H image (C) displays a more homogeneous signal distribution across the entire body compared to the uncorrected image (A). In contrast, for ^23^Na MRI we used a synthetic prior image, based on anatomical ^1^H segmentations (B). The estimated ^23^Na B_1_ bias field (D) exhibits a smooth profile, which does not contain anatomical structures and only corrects the low‐frequency variations of the B_1_ bias field arising from B_1_^+^ and B_1_^−^ inhomogeneities. The B_1_ corrected ^23^Na image (C) shows a more homogeneous ^23^Na signal distribution across the heart.

### 3.5 Quantification and Repeatability Study

**FIGURE 9:**
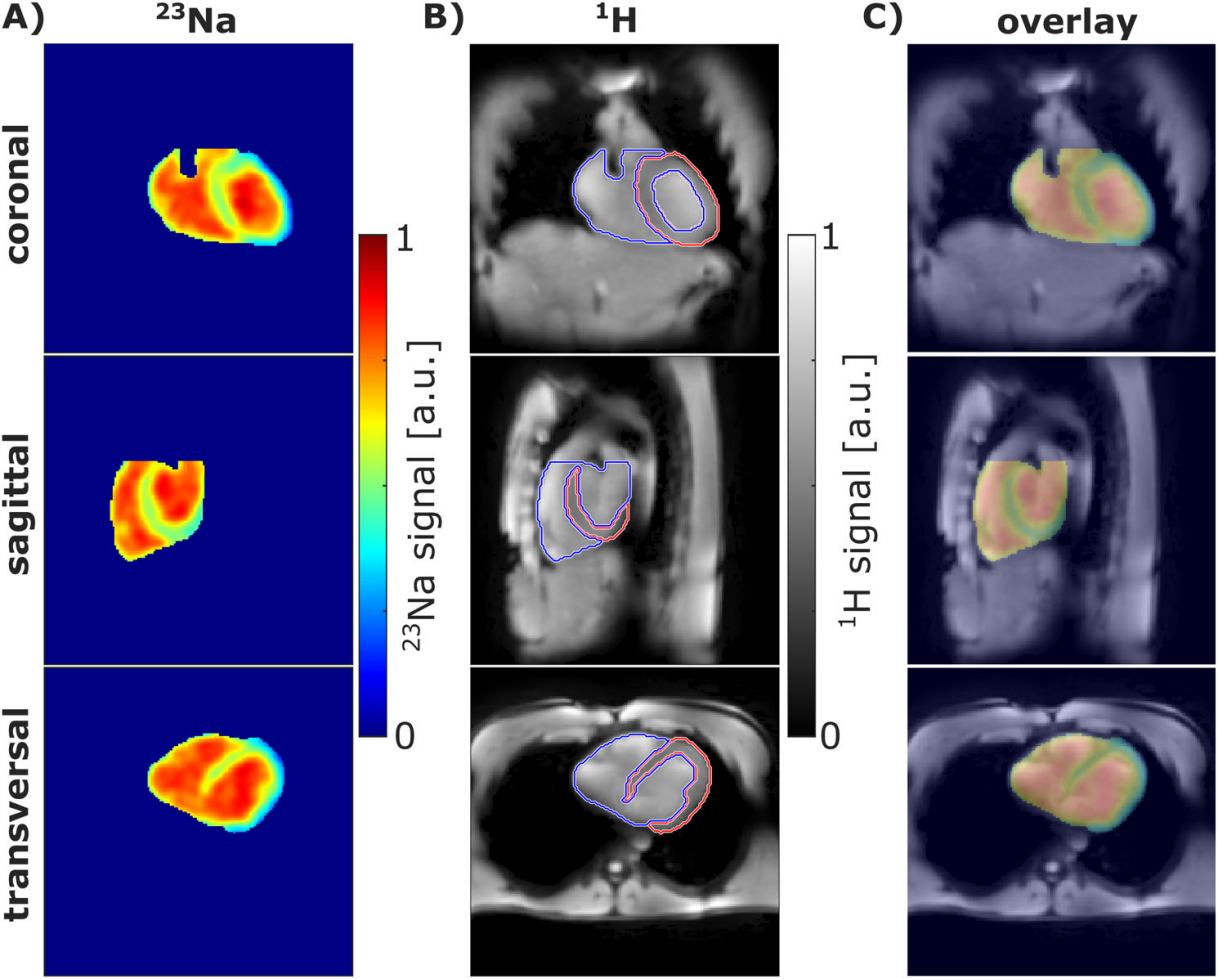
Overview of interleaved ^23^Na and ^1^H MRI of one subject, after application of respiratory and cardiac motion correction as well as B_1_ corrections. Since the ^23^Na B_1_ bias field correction was performed only within the heart region, the B_1_ corrected ^23^Na image (A) was masked accordingly. ^1^H images (B) are overlaid with myocardium (red) and blood pool (blue) segmentations predicted by the nnUNet (C). Note that the blood masks are constrained to the uppermost voxel of the myocardium in SI direction (cut‐off visible in coronal and sagittal view). Due to the interleaved acquisition scheme, both ^23^Na and ^1^H MR images are intrinsically co‐registered.

Figure 9 shows the motion‐ and B_1_‐corrected ^23^Na and ^1^H images of one subject, along with the corresponding segmentations of myocardium and blood pool predicted by the nnUNet. Due to the interleaved acquisition scheme, both images are intrinsically aligned.

**FIGURE 10:**
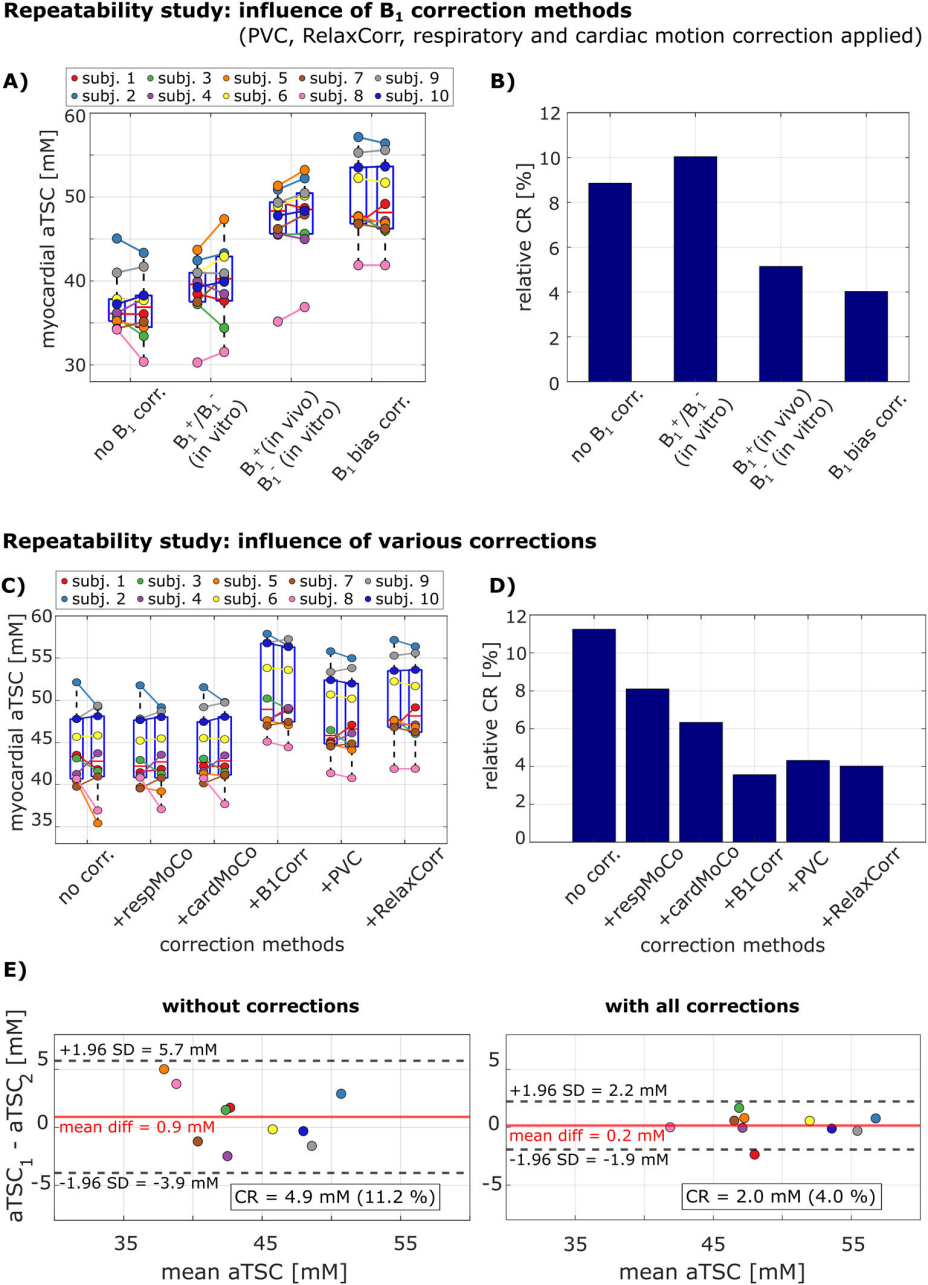
Repeatability study: Influence of B_1_ correction methods (A, B) applied in combination with respiratory and cardiac motion correction as well as PVC and relaxation correction. The aTSC values of measurement 1 and 2 are shown in different box plots for each correction method (A). While the use of in vivo B_1_^+^ maps improved repeatability, the proposed anatomy‐based B_1_ bias field correction achieved the best repeatability (B). Myocardial aTSC values of the in vivo repeatability study after application of the applied correction methods (C). For B_1_ correction, the proposed B_1_ bias field method was used. Each correction method is applied cumulatively from left to right, with each additional method building upon the previous ones. B_1_ bias field correction and partial volume correction had the largest impact on the quantitative aTSC values, while respiratory and cardiac motion correction resulted in smaller aTSC changes. The coefficients of repeatability (D) show that respiratory and cardiac motion correction, the proposed B_1_ correction and the B_1_^+^ dependent relaxation correction improved repeatability, while PVC led to a slight reduction. The overall improvement in repeatability is also visible in the Bland–Altman plots of the aTSC values for no corrections and all correction applied (E).

Figure 10B compares the repeatability of different B_1_ correction methods (Figure S7), with all other corrections applied consistently. Using phantom‐derived B_1_^+^/B_1_^−^ maps resulted in worse repeatability (rCR = 10.0%) than no correction (rCR = 8.9%). A subject‐specific in vivo B_1_^+^ map combined with the phantom B_1_^−^ map improved rCR to 5.1%, though outliers were observed (e.g., subject 8, Figure 10A). The best repeatability was achieved using the proposed anatomy‐based bias field correction (rCR = 4.0%).

Figure 10C shows subject‐wise myocardial aTSC values from the repeatability study (Table S3). Respiratory motion correction impacted aTSC in only a few subjects (e.g., 5 and 9), while cardiac motion correction had a small effect overall. In contrast, B_1_ bias correction increased mean aTSC by 16%. PVC led to a reduction of 6%, while relaxation correction slightly increased it by 3%. After all corrections, the mean myocardial aTSC across all subjects was 49.5 ± 4.7 mM.

Figure 10D summarizes the impact of each correction step on repeatability. Without any corrections, the CR was 4.9 mM (rCR = 11.2%). Applying respiratory motion correction reduced the CR to 3.5 mM (rCR = 8.1%), and adding cardiac motion correction further improved it to 2.8 mM (rCR = 6.3%). The most substantial improvement resulted from B_1_ bias field correction, lowering the CR to 1.8 mM (rCR = 3.6%). PVC caused a slight increase to 2.1 mM (rCR = 4.3%), while subsequent relaxation correction yielded a final CR of 2.0 mM (4.0%) with a 95% confidence interval of 1.4–3.7 mM (rCR = 2.8%–7.3%). The overall improvement in repeatability is also illustrated in the Bland–Altman plots (Figure 10E).

## 4 Discussion

In this work, we demonstrated the feasibility of retrospective respiratory and cardiac motion correction combined with a novel anatomy‐based B_1_ bias field correction for interleaved ^23^Na/^1^H cardiac MRI at 7 T. These methods significantly improved the repeatability of myocardial aTSC quantification.

### 4.1 Respiratory and Cardiac Motion Correction

Breath‐holding is not feasible in ^23^Na MRI due to low SNR and long scans, so respiratory motion is usually recorded and data is retrospectively gated using external devices like respiratory belts [10]. Conventional ECG‐based cardiac gating is unreliable at 7 T due to magnetohydrodynamic effects, though acoustic gating [7] can mitigate this at the cost of additional hardware.

Respiratory and cardiac motion can be extracted directly from ^1^H MR data without external devices [40]. In ^23^Na MRI, self‐gating has mainly been applied for respiratory motion [10], while cardiac gating remains challenging due to low SNR. To overcome this, we incorporated ^1^H navigators into the interleaved sequence. These navigators enabled respiratory and cardiac self‐gating from ^1^H data, which could be directly applied to the ^23^Na data due to the interleaved acquisition. This achieved finer respiratory resolution than prior ^23^Na MRI studies [10] and successfully incorporated cardiac self‐gating for ^23^Na. However, gating reduces acquisition efficiency and SNR, being particularly problematic for ^23^Na MRI.

Instead, we applied retrospective respiratory and cardiac motion correction, fully utilizing all ^23^Na data. Extending prior work correcting rigid head movements in brain ^23^Na/^1^H data [11], respiratory motion was corrected in k‐space assuming rigid heart motion. In contrast, cardiac motion was addressed through nonrigid registration of diastolic and systolic ^1^H images, with corresponding deformation fields applied to ^23^Na images.

Simulations confirmed that the implemented respiratory and cardiac motion corrections reduced quantitative errors and improved SNR compared to motion gating. In vivo, respiratory motion correction decreased quantitative errors and especially preserved ^1^H image quality during deep breathing (Figure 6A). The SNR gain over gating was confirmed, and measured respiratory displacements (Table S3) matched literature values [41, 42]. While cardiac motion correction had a smaller quantitative impact, it nonetheless contributed to improved repeatability.

Overall, this integrated motion correction strategy represents a significant advance, enabling more reliable and efficient myocardial sodium quantification at 7 T. By fully leveraging interleaved ^23^Na/^1^H data and avoiding the SNR penalties of gating, our method paves the way for improved clinical research applications of cardiac ^23^Na MRI.

### 4.2 B_1_ Correction

Myocardial TSC quantification is also affected by the nonuniform B_1_ field distribution of the RF coil used in this work [8]. While the B_1_^+^ transmission field can be measured in vivo, the B_1_^−^ receive field is typically estimated via phantom scans or electromagnetic field simulations based on representative body models [43], both potentially introducing errors.

For B_1_ bias correction of the qualitative ^1^H MRI, we assumed a homogeneous support region following previous works that treated the organ signal as uniform [32, 33]. While this B_1_ correction is simple, fast, and relatively robust, it reduces the contrast between myocardium and blood. However, for our application, the quality of the B_1_ corrected ^1^H images was sufficient to enable nnUNet based segmentation as well as estimation of the motion fields for cardiac motion correction.

To address B_1_ inhomogeneities in quantitative ^23^Na MRI, we proposed a novel anatomy‐based B_1_ bias field correction approach. Our method aims to estimate the low‐frequency B_1_ bias field by comparing the measured ^23^Na image with a synthetic one.

However, unlike the previous method assuming a homogenous support region, we incorporate anatomical information: Segmentations from interleaved ^1^H images are used to synthesize a ^23^Na image with assumed constant TSCs, including PSF convolution. Low‐pass filtering of both the original and synthetic images then isolates the B_1_ bias field.

With the true myocardial sodium concentration unknown, we iteratively tested assumed myo‐blood ratios. B_1_ correction and PVC were applied for each, selecting the assumed ratio best matching post‐correction measured ratio as optimal. Simulations confirmed effective B_1_ correction across varied simulated myocardial‐to‐blood TSC ratios and B_1_ fields (Figure 5). However, PVC errors may affect the selection of the optimal ratio and thus B_1_ estimates. Another limitation is the reliance on accurate anatomical segmentations. Although Gaussian filtering reduces high‐frequency errors, segmentation inaccuracies can still affect the correction (Figure S6—A/BII). Nevertheless, applying erosion or dilation to the in vivo myocardial segmentations had little effect on repeatability and thus on the performance of the in vivo B_1_ correction (Figure S8).

Lacking electromagnetic simulations for the coil used, we compared our method to two alternatives: (1) phantom‐derived B_1_^−^ and B_1_^+^ maps and (2) phantom B_1_^−^ combined with in vivo B_1_^+^ maps. Phantom‐based correction performed worse than no correction, highlighting the importance of subject‐specific B_1_ correction. Using subject‐specific B_1_^+^ maps improved repeatability but remained inferior to our method. This may be because our method accounts for subject‐specific anatomy and coil positioning—both of which affect the B_1_ field distribution—whereas using a phantom‐based B_1_^−^ map does not. Applying the AFI method to measure the ^23^Na B_1_^+^ maps in vivo results in minor deviations of the measured B_1_^+^ field depending on the *T*_1_ relaxation time of the tissue (Figure S9A). While an additional correction of these effects led to a slight reduction of the quantified aTSC values, the coefficients of repeatability were not influenced (Figure S9B). While the AFI method was suited to be a good choice for the application in the human torso, depending on the application, other B_1_^+^ mapping methods might be beneficial [12].

In summary, the proposed method outperforms the other tested methods in terms of repeatability. Moreover, it eliminates the need for additional B_1_^+^ mapping—provided the flip angle has negligible influence on the ^23^Na T_1_ relaxation correction (Equation 1). Additionally, it avoids the need for a specialized phantom with body‐matched dielectric properties to measure the B_1_^−^ field, which may not be available at all sites.

### 4.3 Quantification and Repeatability

Lott et al. [7] quantified the myocardial TSC in four healthy subjects using respiratory and cardiac gating, B_0_ and B_1_ correction, as well as PVC. Similarly, cardiac and respiratory motion gating/correction had minor effects on aTSC values in our study. However, our ^23^Na volume coil [8] exhibits greater B_1_ field inhomogeneity than theirs [10], which likely explains the larger impact of the B_1_ correction observed here. Additionally, PVC reduced myocardial mean aTSC by only 6% in our work, compared to 30% reported by Lott et al. This discrepancy may be partly attributed to errors in the affine coregistration between the ^1^H and ^23^Na images acquired at 3 and 7 T, respectively. Our interleaved acquisition scheme intrinsically aligns ^23^Na and ^1^H images, avoiding such registration issues. Inaccuracies in the nnUNet‐predicted masks used in this study may also have contributed to the observed differences (Figure S8). Regarding the interleaved acquisition scheme, phantom measurements showed no relevant influence on the ^23^Na MR signal [8].

In our study, a mean myocardial aTSC of 49.5±4.7mM was measured for the entire myocardium of the left ventricle. This is in good accordance with extracellular and intracellular volume based estimates (43mM [1]) as well as measured values reported by Ouwerkerk et al. [44] (free wall: 45±4mM, septum: 56±13mM), which were calculated from the original concentrations in μmol/g using a specific gravity of 1.05g/mL for myocardium [44]. Lott et al. reported a lower value of 29±3mM. In addition to coregistration errors, this discrepancy may also be partially caused by residual relaxation weighting due to their shorter TR of 21 ms. Notably, the exact myocardial aTSC values are currently unknown, and our results further demonstrate that segmentation inaccuracies (Figure S6) and deviations in true relaxation times (Figure S5) can influence quantification.

To further improve segmentation consistency, we trained a nnUNet to predict myocardium and blood pool masks using both contrasts. This approach enabled automatic segmentation of motion‐corrected images of the training data and may reduce inter‐reader variability, indicated by a high average DICE similarity score between the segmentations of both measurements (Figure S10). Future work should evaluate the nnUNet's performance on unseen data and expand training to include patient datasets.

Evaluation of the in vivo repeatability study showed that the implemented respiratory and cardiac motion correction combined with the proposed B_1_ correction substantially improved repeatability. PVC slightly reduced it, likely because its segmentation‐derived correction factors amplify segmentation differences. After all corrections, myocardial aTSC variation, caused by measurement noise and post‐processing, is expected to remain below 2.0 mM with 95% confidence. In addition, the time‐efficient interleaved sequence reduced the total scan time to 18 min—significantly shorter than previous 7 T cardiac ^23^Na MRI protocols requiring at least 47 min [7, 43]. Together, the achieved precision and reduced scan time support reliable application in future patient studies.

### 4.4 Limitations

This study has several limitations. Cardiac motion correction was restricted to two phases (diastole and systole), which may not fully capture the complexity of cardiac motion. Advanced reconstruction techniques [45, 46] reducing undersampling artifacts could enable the inclusion of more cardiac phases, further mitigating cardiac motion effects. If working properly, the usage of a simultaneously logged ECG signal for the cardiac binning could improve binning precision, as the ECG provides a higher temporal resolution of 2.5 ms compared to the ≈15 ms of the self‐gated cardiac navigator. Moreover, respiratory motion was assumed rigid, despite containing nonrigid components. Future approaches incorporating nonrigid respiratory models may further improve the correction. Patients may show irregular breathing and heart rates. While respiratory motion can be extracted and corrected regardless of regularity (Figure S11), cardiac irregularities may require adapting the subject‐specific Butterworth filter or using a broader cardiac frequency window (0.5–2 Hz). For severe heart‐rate variations, cardiac binning could alternatively rely on ECG or pulse‐meter signals.

The proposed ^23^Na B_1_ bias correction assumed a homogeneous myocardial sodium concentration, which is reasonable for conditions such as Conn syndrome or chronic kidney disease. In myocardial infarction, however, infarcted tissue has altered sodium levels, which may introduce errors in the B_1_ bias correction. In such cases, we recommend extending the algorithm to use three segmentations—healthy myocardium, infarcted myocardium, and blood—and to optimize both corresponding myo‐blood ratios.

PVC only accounted for the myocardium and blood pool, omitting surrounding tissues. Although Lott et al. [7] suggested that their influence is minor, this simplification may still introduce small quantification errors. The 2 mm isotropic spatial resolution of the interleaved ^1^H MRI may also limit the mitigation of partial volume effects.

Due to specific absorption rate and RF power constraints, a relatively long TE of 1.15 ms had to be used resulting in partial signal loss of the short *T*_2_* component. Moreover, the TR of 60 ms introduced residual *T*_1_ weighting. Both relaxation effects were corrected using literature values; however, deviations from the assumed relaxation times can lead to errors (Figure S5—II). Thus, future studies could improve accuracy by measuring *T*_2_* and *T*_1_ in vivo. Theoretically, quantification may be affected by additional spin‐3/2 specific relaxation effects during the relatively long RF pulse of 2 ms [31, 36]. However, spin‐3/2 dynamic simulation [47, 48] for the assumed relaxation times showed that the resulting transverse magnetization at TE = 1.15 ms depends only marginally on the RF pulse characteristics. Moreover, relaxation effects are adequately corrected by the applied biexponential relaxation model. Nevertheless, excitation‐related signal losses may become more significant in tissues with shorter relaxation times and/or quadrupolar splitting and should be investigated for the specific application.

## 5 Conclusion

This work presents a comprehensive framework for quantitative myocardial ^23^Na MRI using a dual‐nuclear interleaved ^23^Na/^1^H sequence at 7 T. Respiratory and cardiac motion correction were implemented based on the interleaved ^1^H data to retrospectively correct the ^23^Na images. Additionally, we introduced a novel anatomy‐based ^23^Na B_1_ bias field correction using ^1^H‐derived segmentations. These corrections substantially improved measurement repeatability (CR = 2.0 mM, rCR = 4.0%) and yielded a myocardial aTSC of 49.5±4.7mM, consistent with values reported in literature. Combined with the time‐efficient interleaved acquisition and automated segmentation using a nnUNet model, this pipeline provides a robust and scalable foundation for patient‐specific myocardial sodium quantification.

## Funding

This work was supported by Deutsche Forschungsgemeinschaft (449552397, 509149993) (TRR 374).

## Supporting information


**Table S1:** Tissue parameters for ^23^Na simulation. Assumed values are marked by (*). The myocardial TSC of 43 mM was derived from 41 μmol/g [1] by applying a specific gravity of 1.05g/mL for myocardium [44].
**Table S2:** Tissue parameters for ^1^H simulation. Assumed values are marked by (*).
**Figure S1:** Comparison of cardiac self‐gating signal (SG), derived from the interleaved ^1^H data, and simultaneously acquired electrocardiogram (ECG) for three subjects. Due to the filtering in the post‐processing of the SG signal, the triggers of SG tend to occur in mid‐diastole, while scanner‐predicted ECG triggers (M1, red circles) mark the end of the diastolic phase. Additionally, the minima of the ECG signal were used as triggers (M2, blue circles). For subject 6 (a), the histogram of the temporal difference ΔT between consecutive triggers showed good agreement between SG and ECG independent of the selected triggers. However, for subjects 7 (b) and 8 (c) scanner‐based ECG triggering (M1) resulted in trigger errors. In contrast, minima based ECG trigger (M2) and SG showed consistent triggering for all three subjects.
**Figure S2:** Influence of the standard deviation σ of the Gaussian low‐pass filter in the ^23^Na B_1_ bias correction (see Figure S3). For this optimization, the phantom B_1_ bias field and the simulated ^23^Na GT of a simulation with myocardial TSC of 43 mM (see top row of Figure 5A) was used and Gaussian noise was added. In this case, the assumed myo‐blood ratio for the ^23^Na prior information $r_{\text{assumed}}$ was exactly set to match the simulation. Then, the in Figure S3 described procedure was performed using different σ in the low‐pass filtering step. Finally, the obtained B_1_ corrected ^23^Na images were compared to the ^23^Na GT via NRMSE. Too low σ does not suppress noise sufficiently, while too high σ loses some of the spatial low‐frequency components of the B_1_ bias field. The optimal σ was found for σ = 10 mm, which was used for the ^23^Na B_1_ bias correction.
**Figure S3:** Overview of the implemented B_1_ bias field correction for ^1^H and ^23^Na MRI. For ^1^H MRI (A) a homogeneous support region was created by thresholding. The B_1_ uncorrected ^1^H image and the supporting region were then low‐pass filtered using a Gaussian filter with standard deviation $\sigma_{1H}=12\;\mathrm{mm}$ and divided to obtain the estimated ^1^H B_1_ bias field. The B_1_ uncorrected ^1^H image was divided by the estimated ^1^H B_1_ bias field resulting in a B_1_ corrected ^1^H image. Using a support region as anatomical prior leads to some errors, however, since ^1^H images are only used for segmentation minor errors can be neglected. In contrast, for ^23^Na MRI (B) the algorithm was designed more complex to reduce quantitative errors. Here, the ^1^H based segmentations of myocardium and blood pool were convoluted with the *T*_2_* dependent corresponding point‐spread functions (PSF) to obtain region‐spread functions (RSF) of both tissues. These RSF were then summed up to the prior information $r_{\text{assumed}}\cdot \mathrm{RS}F_{\mathrm{myo}}+\mathrm{RS}F_{\text{blood}}$ using an assumed myo‐blood ratio $r_{\text{assumed}}$. Subsequently, the B_1_ uncorrected ^23^Na image and the ^23^Na prior information were masked and Gaussian filtered with $\sigma_{23\mathrm{Na}}=10\;\mathrm{mm}$. Division of both yields the estimated ^23^Na B_1_ bias field, which was used to correct the B_1_ uncorrected ^23^Na image within the heart region, yielding a B_1_ corrected ^23^Na image. Partial volume correction—based on the mean signals in myocardium and blood—of the B_1_ corrected ^23^Na image allows to quantify the myo‐blood ratio $r_{\text{measured}}$ after the application of the B_1_ bias correction, which is then compared to the input $r_{\text{assumed}}$. The entire procedure is repeated iteratively for different $r_{\text{assumed}}$ and the one showing the lowest absolute difference of $r_{\text{assumed}}$ and $r_{\text{measured}}$ is selected as the optimal choice.
**Figure S4:** Comparison of the double angle and actual flip‐angle imaging method for ^23^Na B_1_^+^ mapping in one healthy subject. The double angle method [5] was based on the acquisition of two images with FA = 45° and FA = 90° using a TR = 250 ms to ensure full *T*_1_ relaxation. The AFI method used a FA = 60° and TR1 = 12 ms and TR2 = 48 ms. For both methods the total acquisition time was set to 15 min. The outline of the heart was drawn on an additional ^1^H image (A). The FA determined using the DAM (B) is higher compared to the AFI method (C). This is likely caused by the *T*_1_ bias of the AFI sequence. In general, the AFI appears smoother and showed no high‐frequency FA variations within the heart. When accelerating the AFI measurement from 15 to 3 min, an additional Gaussian filter (imgaussfilt3 in Matlab) with standard deviation of 14 mm was applied to the FA map. The resulting FA map (D) showed a similar FA distribution as for the 15 min AFI measurement (C).
**Figure S5:** Influence of deviations of the myocardial T*_2,short_ to the assumed value of 3 ms. The influence was evaluated for the B_1_ bias correction (I) as well as entire quantification procedure, including B_1_ bias correction, partial volume correction and relaxation correction. Simulations were performed for a myocardial TSC of 43 mM and myocardial T*_2,short_ times, which were shorter (2.4 ms) or longer (3.6 ms) than or the same as the 3 ms assumed for all corrections. The B_1_ bias correction was evaluated for three different B_1_ bias field distributions as shown in Figure 5. B_1_ bias correction was almost not influenced by the deviating T*_2,short_ times as B_1_ corrected images showed low and stable aTSC deviations relative to the B_1_ free GT images and lower deviations than the B_1_ uncorrected images across all three B_1_ distributions and all three simulated T*_2,short_ times (I). When considering the entire quantification procedure, stronger aTSC deviations are visible, mainly caused by errors in the performed relaxation correction of the *T*_2_* decay. Over all three simulated T*_2,short_ values, the quantified aTSC showed deviations between −23.4% and +17.1% for the B_1_ uncorrected images, while this reduced to −7.4% and 12% after B_1_ correction.
**Figure S6:** Influence of imperfect segmentations for normal (A) as well as hypertrophied heart (B), which was additionally simulated. In order to evaluate the influence of segmentation errors, we eroded (spherical erosion with one voxel radius) and dilated (spherical dilation with one voxel diameter) the original myocardial masks. This resulted in an increase/decrease of the myocardial volume of −/+25% and −/+14% for the normal and hypertrophied heart, respectively (I). The ^23^Na B_1_ bias correction was evaluated for the three different B_1_ bias field distributions as shown in Figure 5. For both normal and hypertrophied heart, a thinner segmented myocardium results in a slight overcorrection when applying the B_1_ correction, while a too thick segmented myocardium leads to a slight undercorrection (II). Over all three used mask configurations and B_1_ bias field distributions, for the normal heart (A) the quantified aTSC showed deviations between −22.9% and +14.5% for the B_1_ uncorrected images, which were reduced to −4.3% and +10.5% after B_1_ correction (III). For the hypertrophied heart (B), deviations for the B_1_ uncorrected images ranged between −15.5% and +11.5% and between −8.4% and +6.8% after B_1_ correction.
**Table S3:** Quantitative results of the repeatability study in 10 healthy subjects. Listed are subject‐specific characteristics (gender, age, height, weight, BMI), physiological parameters derived during reconstruction (respiratory and cardiac frequency, cardiac peak width, maximal respiratory displacement in the superior–inferior direction), as well as tissue sodium concentrations (aTSC) for blood (measured from blood samples) and myocardium (measured by ^23^Na MRI).
**Figure S7:** Influence of different B_1_ correction methods on ^23^Na MRI. The B_1_ corrections were applied after respiratory and cardiac motion correction. Three different B_1_ correction methods were compared: (1) B_1_^+^ and B_1_^−^ field measured in phantom; (2) B_1_^+^ field measured in vivo using the AFI sequence and B_1_^−^ field measured in phantom; (3) proposed B_1_ bias field correction. Note that since the B_1_ bias field correction was applied only within the heart, the B_1_ bias corrected ^23^Na image was masked accordingly (D). ^23^Na signal across the blood pool displayed a homogenous signal distribution for the B_1_ bias field correction (D), which is not the case for the other two methods (B, C). Compared to the uncorrected image (A), B_1_ bias field correction increased the ^23^Na signal, in particular in superior left regions of the heart.
**Figure S8:** Influence of myocardial masks size on quantitative myocardial aTSC as well as repeatability. All corrections were applied as in Figure 10C, with B_1_ bias field correction and partial volume correction in particular depending on the segmentations. The original myocardial masks were eroded or dilated using a spherical structuring element with a radius of 1 voxel. The relative change in myocardial mask volume compared to the original mask, averaged across all 10 subjects, was: –30% ± 3% for erosion and +30% ± 3% after dilation. (A) shows an overlay of the modified myocardial (red) and blood pool (blue) masks for subject 1. Reducing the myocardial mask size led to an increase in measured myocardial aTSC values, whereas dilation resulted in a decrease (B). The changes in global mean myocardial aTSC were: +7.6% for the eroded masks and −5.2% for the dilated masks. There were no relevant influences of the masks on the repeatability (C).
**Figure S9:** Influence of the AFI approximation on flip angles measured in myocardium and blood. The AFI method assumes $TR_{1/2}\ll T_{1}$. Since this is not fulfilled for the applied ^23^Na AFI measurements ($TR_{1}=12\;\mathrm{ms},TR_{2}=48\;\mathrm{ms}$), this leads to deviations of the measured flip angle compared to the true flip angle (A). These deviations depend on the *T*_1_ relaxation time of the tissue and are therefore higher in myocardium than in blood. The found relation in (A) allows to correct this effect for the different compartments assuming specific *T*_1_ times ($T_{1,\mathrm{myo}}=30\;\mathrm{ms},T_{1,\text{blood}}=49.5\;\mathrm{ms}$). For the method using the AFI for the estimation of the B_1_^+^ distribution and the B_1_^−^ map from the phantom (left side in B), this reduced the myocardial aTSC on average by 2.2 mM and slightly worsened the repeatability from CR = 5.1% to 5.3%. When applying the B_1_ bias correction (right side in B), the AFI measurement was only used to determine the flip angle for the *T*_1_ relaxation correction. The average aTSC was decreased by −0.9 mM, while the repeatability slightly improved from 4.0% to 3.9%.
**Figure S10:** Assessment of the variability of the segmentations predicted by the nnUNet. For the evaluation of the DICE score the ^1^H image of the second measurement was registered to the ^1^H image of the first measurement. The segmentations of measurement 2 were than transformed accordingly. DICE scores for the myocardial and blood segmentation between both measurements (A) indicate good accordance. The mean DICE score across all subjects and both compartments was 0.89±0.04. Relative volume changes of the myocardial and blood masks (B) ranged between −11.1% and 9.1%.
**Figure S11:** Validation of respiratory motion correction for different breathing patterns. In this measurement of one healthy subject (male, 28 years, 83 kg), the subject was instructed to change breathing patterns: (A) normal breathing, (B) shallow breathing, (C) deep breathing, (D) random breathing. Each pattern was performed for around 3:45 min. The ^1^H based self‐gating navigator signal (top) captures all four breathing patterns and exhibits amplitude variations associated with different breathing depths. For the entire measurement the respiratory motion correction algorithm was applied as described in the paper, such that all acquired projections were binned into eight different respiratory bins and corrected into the fully‐exhaled state. Here, bin 1 corresponds to the fully‐inhaled state, while bin 8 represent the fully‐exhaled state. For better assessment of the respiratory motion correction for the different breathing patterns, uncorrected and respiratory motion‐corrected ^23^Na and ^1^H images of each phase (A–D) were reconstructed as well as of the entire measurement (E). Note that the shown images do not contain cardiac motion correction and B_1_ correction was only applied for ^1^H, not ^23^Na MRI. Applying respiratory motion correction, ^23^Na and ^1^H images of all phases are aligned in the fully‐exhaled state, while motion artifacts are especially visible in the uncorrected images for deep and random breathing. Differences between uncorrected and respiratory corrected images are more pronounced for deep and random breathing compared to normal or shallow breathing as expected.

## Data Availability

The data that support the findings of this study are available on request from the corresponding author. The data are not publicly available due to privacy or ethical restrictions.
