# Supplementary material for: Improved Myocardial Sodium Quantification at 7 T Using Interleaved 23Na/ 1H pTx MRI With Motion and Anatomy‐Based B1 Correction
Source: Magn Reson Med. 2026 Mar 16;96(1):173–90. doi: 10.1002/mrm.70342 (PMC13156449; doi:10.1002/mrm.70342)
Supplement: Supplementary file 1 — Table S1: Tissue parameters for 23Na simulation. Assumed values are marked by (*). The myocardial TSC of 43 mM was derived from 41 μmol/g [1] by applying a specific gravity of 1.05g/mL for myocardium [44]. Table S2: Tissue parameters for 1H simulation. Assumed values are marked by (*). Figure S1: Comparison of cardiac self‐gating signal (SG), derived from the interleaved 1H data, and simultaneously acquired electrocardiogram (ECG) for three subjects. Due to the filtering in the post‐processing of the SG signal, the triggers of SG tend to occur in mid‐diastole, while scanner‐predicted ECG triggers (M1, red circles) mark the end of the diastolic phase. Additionally, the minima of the ECG signal were used as triggers (M2, blue circles). For subject 6 (a), the histogram of the temporal difference ΔT between consecutive triggers showed good agreement between SG and ECG independent of the selected triggers. However, for subjects 7 (b) and 8 (c) scanner‐based ECG triggering (M1) resulted in trigger errors. In contrast, minima based ECG trigger (M2) and SG showed consistent triggering for all three subjects. Figure S2: Influence of the standard deviation σ of the Gaussian low‐pass filter in the 23Na B1 bias correction (see Figure S3). For this optimization, the phantom B1 bias field and the simulated 23Na GT of a simulation with myocardial TSC of 43 mM (see top row of Figure 5A) was used and Gaussian noise was added. In this case, the assumed myo‐blood ratio for the 23Na prior information rassumed was exactly set to match the simulation. Then, the in Figure S3 described procedure was performed using different σ in the low‐pass filtering step. Finally, the obtained B1 corrected 23Na images were compared to the 23Na GT via NRMSE. Too low σ does not suppress noise sufficiently, while too high σ loses some of the spatial low‐frequency components of the B1 bias field. The optimal σ was found for σ = 10 mm, which was used for the 23Na B1 bias correction. Figure S3: Overview of th [file MRM-96-173-s001.docx]

**Supporting Information**

Table S1: Tissue parameters for ²³Na simulation. Assumed values are marked by (*). The myocardial TSC of 43mM was derived from 41$\mu mol/g$ ([1](#_ENREF_1)) by applying a specific gravity of $1.05g/ml$ for myocardium ([2](#_ENREF_2)).

| Tissue | TSC [mM] | T_1_ [ms] | T*_2,short_ [ms] | T*_2,long_ [ms] | r |
| --- | --- | --- | --- | --- | --- |
| blood | 81 ([3](#_ENREF_3)) | 49.5 (venous) ([3](#_ENREF_3)) | - | 14.7 ([3](#_ENREF_3)) | 0 ([3](#_ENREF_3)) |
| myocardium | 43 ([1](#_ENREF_1)) | 30 * (calf) ([3](#_ENREF_3)) | 3 * (calf) ([3](#_ENREF_3)) | 26.6 * (calf) ([3](#_ENREF_3)) | 0.6 * ([3](#_ENREF_3)) |
| muscle | 20 ([3](#_ENREF_3)) | 30 (calf) ([3](#_ENREF_3)) | 3 (calf) ([3](#_ENREF_3)) | 26.6 (calf) ([3](#_ENREF_3)) | 0.6 ([3](#_ENREF_3)) |
| liver | 20.1 ([4](#_ENREF_4)) | 30 * | 3 * | 26.6 * | 0.6 * |
| cortex | 58 ([5](#_ENREF_5)) | 36 ([5](#_ENREF_5)) | 19 ([5](#_ENREF_5)) | 19 ([5](#_ENREF_5)) | 0.6 * |
| medulla | 99 ([5](#_ENREF_5)) | 35.4 ([5](#_ENREF_5)) | 18 ([5](#_ENREF_5)) | 18 ([5](#_ENREF_5)) | 0.6 * |
| cartilage | 215 ([6](#_ENREF_6)) | 20 ([7](#_ENREF_7)) | 0.9 ([7](#_ENREF_7)) | 13.3 ([7](#_ENREF_7)) | 0.34 ([6](#_ENREF_6)) |

Table S2: Tissue parameters for ¹H simulation. Assumed values are marked by (*).

| Tissue | Spin density [a.u.] | T_1_ [ms] | T*_2_ [ms] |
| --- | --- | --- | --- |
| blood | 0.95 ([8](#_ENREF_8)) | 2212 ([9](#_ENREF_9)) | 55 (arterial) ([10](#_ENREF_10)) |
| myocardium | 0.7 ([8](#_ENREF_8)) | 1925 ([11](#_ENREF_11)) | 14.2 ([12](#_ENREF_12)) |
| muscle | 0.7 * | 1864 ([13](#_ENREF_13)) | 22 ([13](#_ENREF_13)) |
| liver | 0.7 * | 1864 * | 22 * |
| cortex | 0.7 * | 2094 ([14](#_ENREF_14)) | 22 * |
| medulla | 0.7 * | 1864 ([14](#_ENREF_14)) | 22 * |
| cartilage | 0.5 * | 1503.5 ([15](#_ENREF_15)) | 15.25 ([15](#_ENREF_15)) |


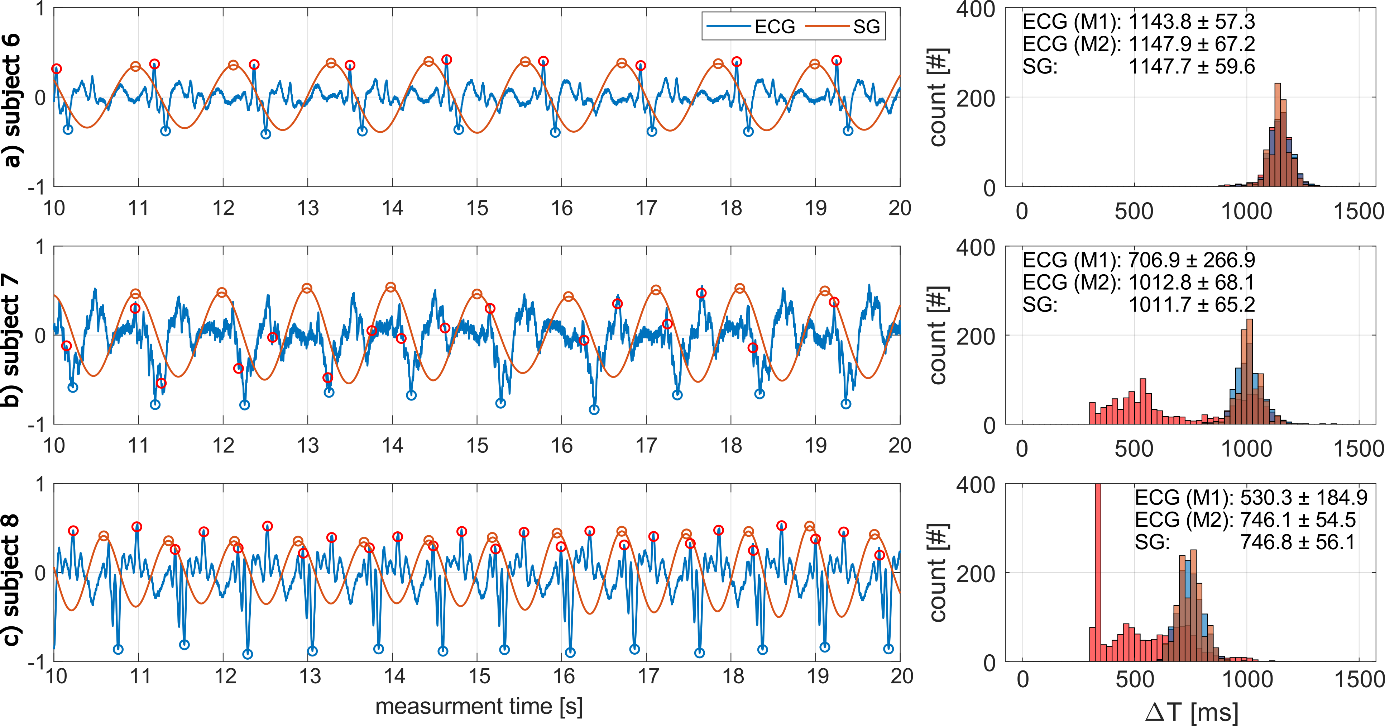


Figure S1: Comparison of cardiac self-gating signal (SG), derived from the interleaved ¹H data, and simultaneously acquired electrocardiogram (ECG) for three subjects. Due to the filtering in the post-processing of the SG signal, the triggers of SG tend to occur in mid-diastole, while scanner-predicted ECG triggers (M1, red circles) mark the end of the diastolic phase. Additionally, the minima of the ECG signal were used as triggers (M2, blue circles). For subject 6 (a), the histogram of the temporal difference $\Delta T$ between consecutive triggers showed good agreement between between SG and ECG independent of the selected triggers. However, for subjects 7 (b) and 8 (c) scanner-based ECG triggering (M1) resulted in trigger errors. In contrast, minima based ECG trigger (M2) and SG showed consistent triggering for all three subjects.


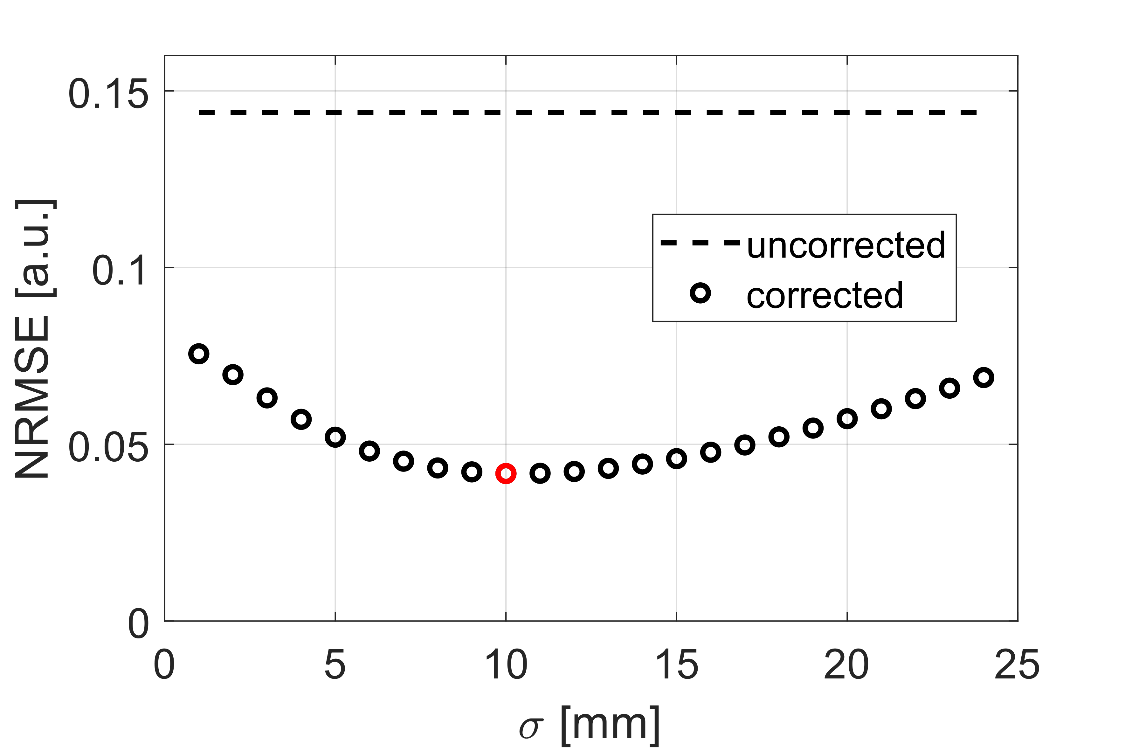


Figure S2: Influence of the standard deviation $\sigma$ of the Gaussian low-pass filter in the ^23^Na B_1_ bias correction (see Figure S3). For this optimization, the phantom B_1_ bias field and the simulated ^23^Na GT of a simulation with myocardial TSC of 43 mM (see top row of Figure 5A) was used and Gaussian noise was added. In this case, the assumed myo-blood ratio for the ^23^Na prior information $r_{assumed}$ was exactly set to match the simulation. Then, the in Figure S3 described procedure was performed using different $\sigma$ in the low-pass filtering step. Finally, the obtained B_1_ corrected ^23^Na images were compared to the ^23^Na GT via NRMSE. Too low $\sigma$ does not suppress noise sufficiently, while too high $\sigma$ loses some of the spatial low-frequency components of the B_1_ bias field. The optimal $\sigma$ was found for $\sigma$=10 mm, which was used for the ^23^Na B_1_ bias correction.


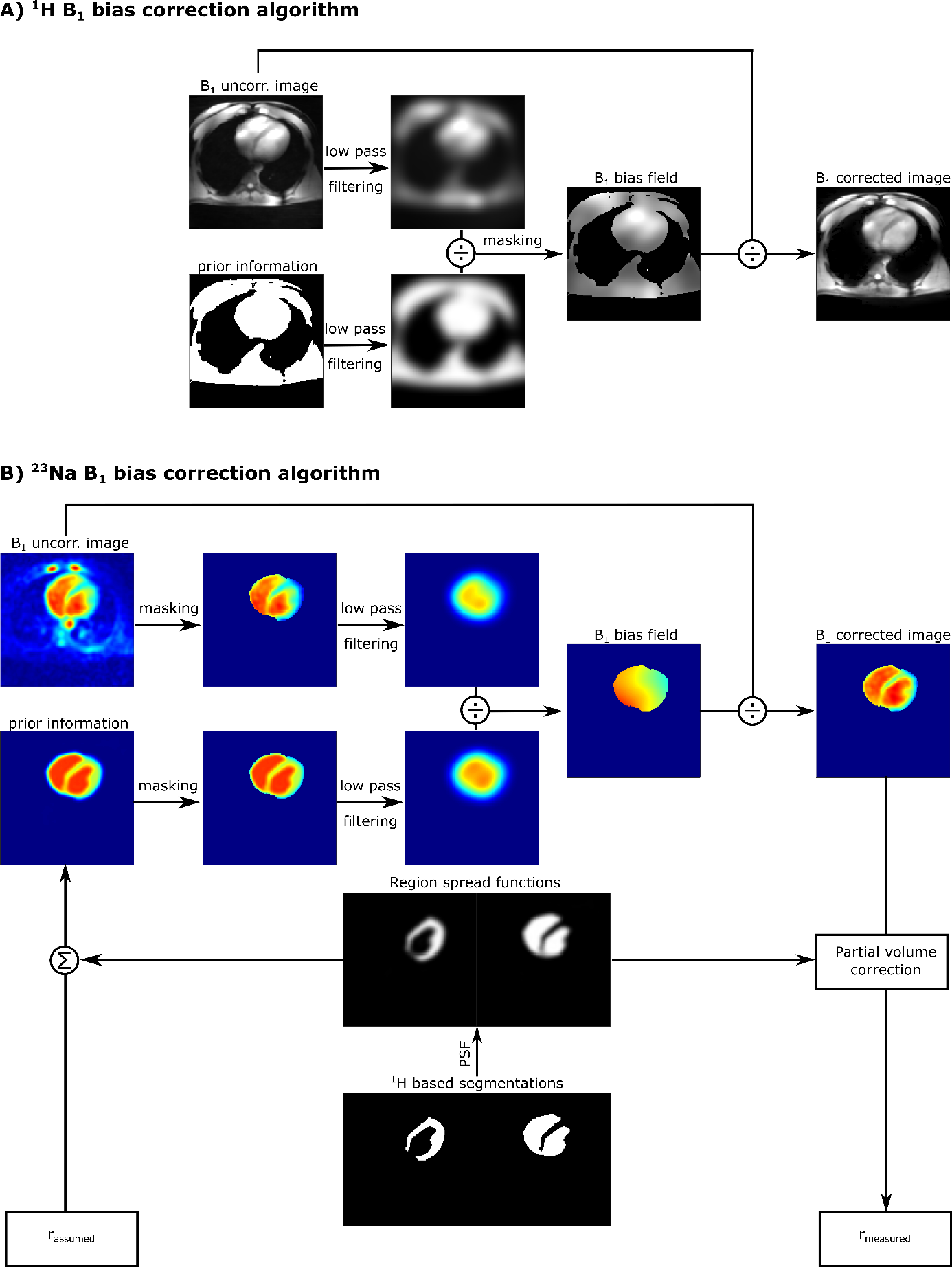


Figure S3: Overview of the implemented B_1_ bias field correction for ^1^H and ^23^Na MRI. For ^1^H MRI (A) a homogeneous support region was created by thresholding. The B_1_ uncorrected ^1^H image and the supporting region were then low-pass filtered using a Gaussian filter with standard deviation $\sigma_{1H}=12 mm$ and divided to obtain the estimated ^1^H B_1_ bias field. The B_1_ uncorrected ^1^H image was divided by the estimated ^1^H B_1_ bias field resulting in a B_1_ corrected ^1^H image. Using a support region as anatomical prior leads to some errors, however, since ^1^H images are only used for segmentation minor errors can be neglected. In contrast, for ^23^Na MRI (B) the algorithm was designed more complex to reduce quantitative errors. Here, the ^1^H based segmentations of myocardium and blood pool were convoluted with the T_2_* dependent corresponding point-spread functions (PSF) to obtain region-spread functions (RSF) of both tissues. These RSF were then summed up to the prior information $r_{assumed}\cdot RSF_{myo}+RSF_{blood}$ using an assumed myo-blood ratio $r_{assumed}$. Subsequently, the B_1_ uncorrected ^23^Na image and the ^23^Na prior information were masked and Gaussian filtered with $\sigma_{23Na}=10 mm$. Division of both yields the estimated ^23^Na B_1_ bias field, which was used to correct the B_1_ uncorrected ^23^Na image within the heart region, yielding a B_1_ corrected ^23^Na image. Partial volume correction – based on the mean signals in myocardium and blood – of the B_1_ corrected ^23^Na image allows to quantify the myo-blood ratio $r_{measured}$ after the application of the B_1_ bias correction, which is then compared to the input $r_{assumed}$. The entire procedure is repeated iteratively for different $r_{assumed}$ and the one showing the lowest absolute difference of $r_{assumed}$ and $r_{measured}$ is selected as the optimal choice.


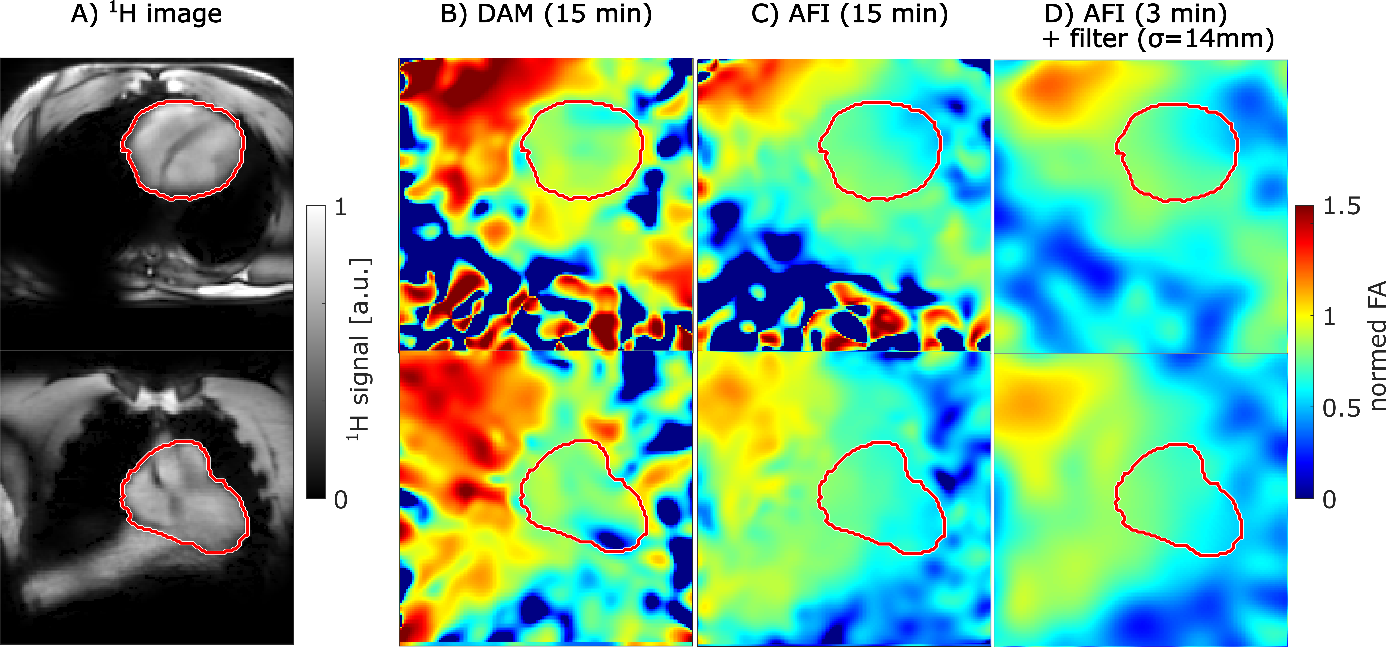


Figure S4: Comparison of the double angle and actual flip angle imaging method for ^23^Na B_1_^+^ mapping in one healthy subject. The double angle method([16](#_ENREF_16)) was based on the acquisition of two images with FA = 45° and FA = 90° using a TR=250ms to ensure full T_1_ relaxation. The AFI method used a FA=60° and TR1 =12 ms and TR2 = 48 ms. For both methods the total acquisition time was set to 15 minutes. The outline of the heart was drawn on an additional ^1^H image (A). The FA determined using the DAM (B) is higher compared to the AFI method (C). This is likely caused by the T_1_ bias of the AFI sequence. In general, the AFI appears smoother and showed no high-frequency FA variations within the heart. When accelerating the AFI measurement from 15 to 3 minutes, an additional Gaussian filter (imgaussfilt3 in Matlab) with standard deviation of 14mm was applied to the FA map. The resulting FA map (D) showed a similar FA distribution as for the 15 minutes AFI measurement (C).


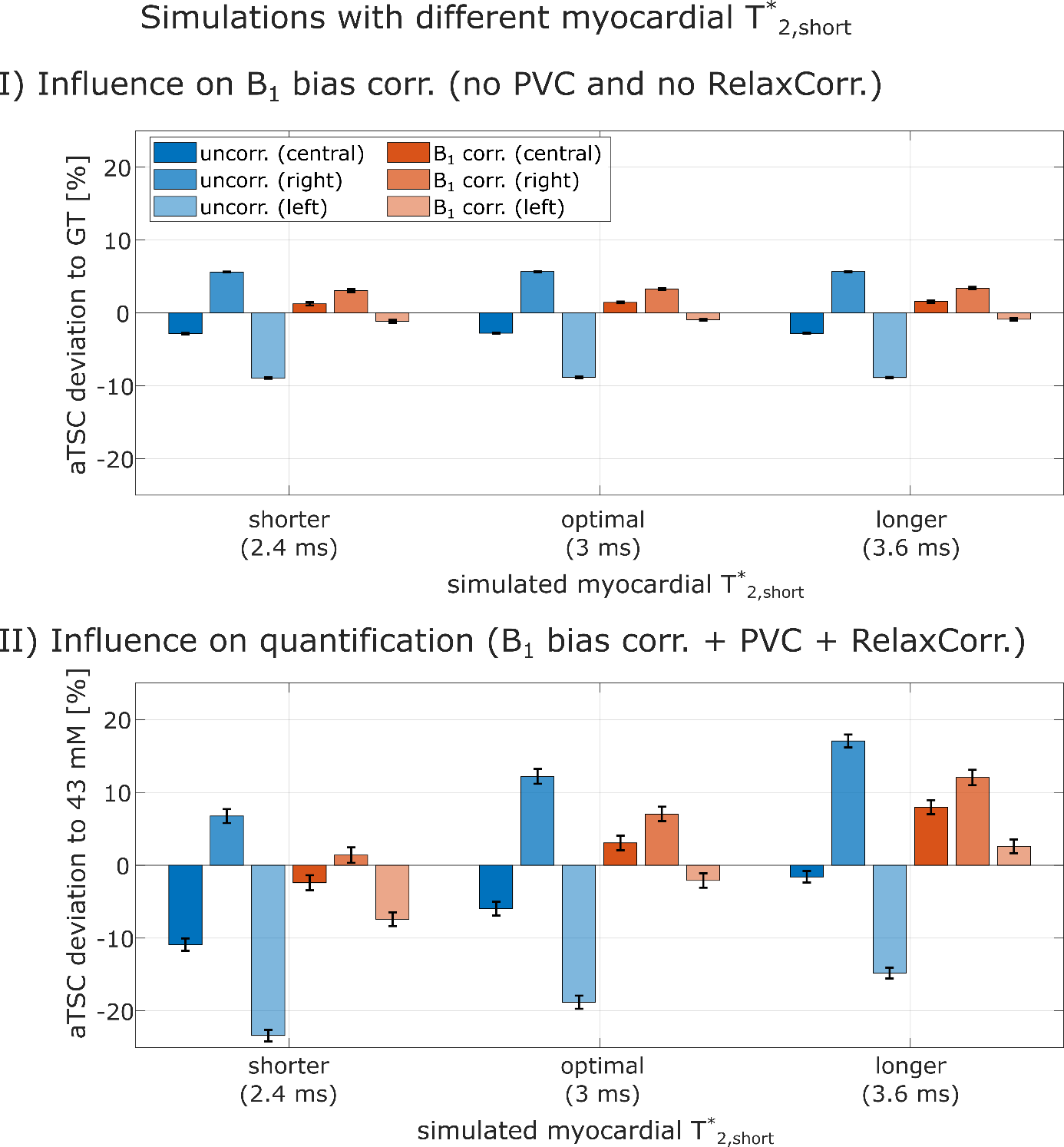


Figure S5: Influence of deviations of the myocardial T*_2,short_ to the assumed value of 3 ms. The influence was evaluated for the B_1_ bias correction (I) as well as entire quantification procedure, including B_1_ bias correction, partial volume correction and relaxation correction. Simulations were performed for a myocardial TSC of 43 mM and myocardial T*_2,short_ times, which were shorter (2.4 ms) or longer (3.6 ms) than or the same as the 3 ms assumed for all corrections. The B_1_ bias correction was evaluated for three different B_1_ bias field distributions as shown in Figure 5. B_1_ bias correction was almost not influenced by the deviating T*_2,short_ times as B_1_ corrected images showed low and stable aTSC deviations relative to the B_1_ free GT images and lower deviations than the B_1_ uncorrected images across all three B_1_ distributions and all three simulated T*_2,short_ times (I). When considering the entire quantification procedure, stronger aTSC deviations are visible, mainly caused by errors in the performed relaxation correction of the T_2_* decay. Over all three simulated T*_2,short_ values, the quantified aTSC showed deviations between -23.4% and +17.1% for the B_1_ uncorrected images, while this reduced to -7.4% and 12% after B_1_ correction.


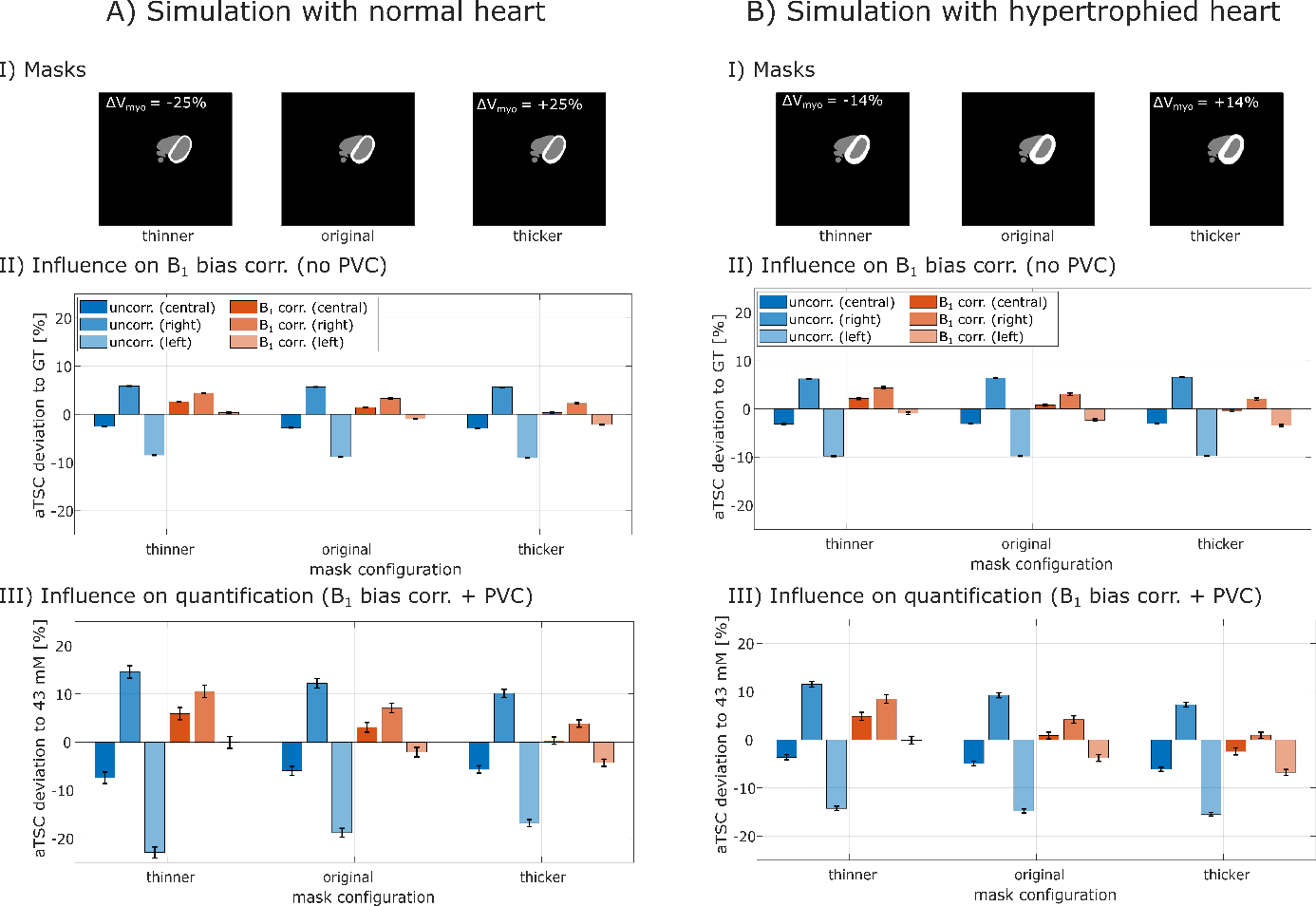


Figure S6: Influence of imperfect segmentations for normal (A) as well as hypertrophied heart (B), which was additionally simulated. In order to evaluate the influence of segmentation errors, we eroded (spherical erosion with one voxel radius) and dilated (spherical dilation with one voxel diameter) the original myocardial masks. This resulted in an increase/decrease of the myocardial volume of -/+25% and -/+14% for the normal and hypertrophied heart, respectively (I). The ^23^Na B_1_ bias correction was evaluated for the three different B_1_ bias field distributions as shown in Figure 5. For both normal and hypertrophied heart, a thinner segmented myocardium results in a slight overcorrection when applying the B_1_ correction, while a too thick segmented myocardium leads to a slight undercorrection (II). Over all three used mask configurations and B_1_ bias field distributions, for the normal heart (A) the quantified aTSC showed deviations between -22.9% and +14.5% for the B_1_ uncorrected images, which were reduced to -4.3% and +10.5% after B_1_ correction (III). For the hypertrophied heart (B), deviations for the B_1_ uncorrected images ranged between -15.5% and +11.5% and between -8.4% and +6.8% after B_1_ correction.

Table S3: Quantitative results of the repeatability study in 10 healthy subjects. Listed are subject-specific characteristics (gender, age, height, weight, BMI), physiological parameters derived during reconstruction (respiratory and cardiac frequency, cardiac peak width, maximal respiratory displacement in the superior-inferior direction), as well as tissue sodium concentrations (aTSC) for blood (measured from blood samples) and myocardium (measured by ²³Na MRI).

| subject characteristics | | | | | |
| --- | --- | --- | --- | --- | --- |
| # subject | **gender** | **age [y]** | **height [cm]** | **weight [k g]** | **BMI [kg/m^2^]** |
|  |  |  |  |  |  |
| 1 | m | 28 | 179 | 83.5 | 26.2 |
| 2 | f | 34 | 174 | 70.6 | 23.3 |
| 3 | m | 26 | 185 | 87.1 | 25.6 |
| 4 | m | 27 | 170 | 74.8 | 26.0 |
| 5 | m | 28 | 188 | 93.3 | 26.4 |
| 6 | m | 25 | 178 | 68.3 | 21.6 |
| 7 | m | 22 | 185 | 91.7 | 26.8 |
| 8 | m | 23 | 170 | 62.6 | 21.8 |
| 9 | f | 30 | 165 | 56.8 | 20.8 |
| 10 | f | 28 | 171 | 68.1 | 23.3 |
| mean |  | 27.1 $\pm$ 3.5 | 176 $\pm$ 8 | 75.7 $\pm$ 12.6 | 24.2 $\pm$ 2.3 |

| physiological characteristics | | | | | | | | |
| --- | --- | --- | --- | --- | --- | --- | --- | --- |
| # | **maximal SI displ [mm]** | | **respiratory frequency [Hz]** | | **cardiac frequency [Hz]** | | **cardiac peak width [Hz]** | |
|  | **meas 1** | **meas 2** | **meas 1** | **meas 2** | **meas 1** | **meas 2** | **meas 1** | **meas 2** |
| 1 | 14.2 | 12.5 | 0.20 | 0.20 | 0.77 | 0.81 | 0.10 | 0.11 |
| 2 | 8.6 | 7.1 | 0.27 | 0.30 | 0.71 | 0.65 | 0.06 | 0.06 |
| 3 | 7.4 | 11.3 | 0.19 | 0.17 | 0.96 | 1.07 | 0.11 | 0.14 |
| 4 | 6.0 | 8.7 | 0.33 | 0.32 | 1.45 | 1.37 | 0.09 | 0.09 |
| 5 | 17.9 | 24.4 | 0.06 | 0.06 | 0.95 | 0.84 | 0.21 | 0.14 |
| 6 | 10.1 | 9.1 | 0.26 | 0.28 | 0.87 | 0.89 | 0.07 | 0.08 |
| 7 | 4.0 | 7.9 | 0.25 | 0.24 | 1.00 | 0.96 | 0.12 | 0.08 |
| 8 | 7.6 | 5.8 | 0.25 | 0.28 | 1.33 | 1.40 | 0.19 | 0.24 |
| 9 | 9.7 | 12.6 | 0.19 | 0.18 | 1.02 | 0.94 | 0.15 | 0.11 |
| 10 | 7.1 | 7.3 | 0.41 | 0.40 | 1.09 | 1.04 | 0.11 | 0.14 |
| mean | 9.6 $\pm$ 4.1 | 10.7 $\pm$ 5.4 | 0.24 $\pm$ 0.09 | 0.24 $\pm$ 0.09 | 1.02 $\pm$ 0.23 | 1.00 $\pm$ 0.21 | 0.12 $\pm$ 0.05 | 0.12 $\pm$ 0.05 |

| measured biomarkers | | | | | |
| --- | --- | --- | --- | --- | --- |
| # | **hematocrit [%]** | **blood TSC [mM]** | **myocardial aTSC [mM]** | | |
|  |  |  | **meas 1** | **meas 2** | **mean** |
| 1 | 44.9 | 76.0 | 46.8 | 49.2 | 48.0 |
| 2 | 38.2 | 84.7 | 57.1 | 56.4 | 56.8 |
| 3 | 40.8 | 82.3 | 47.7 | 46.0 | 46.9 |
| 4 | 46.3 | 76.8 | 47.1 | 47.1 | 47.1 |
| 5 | 43.8 | 78.7 | 47.6 | 46.9 | 47.2 |
| 6 | 39.6 | 85.2 | 52.3 | 51.7 | 52.0 |
| 7 | 45.8 | 74.3 | 46.8 | 46.2 | 46.5 |
| 8 | 46.7 | 71.4 | 41.9 | 41.9 | 41.9 |
| 9 | 39.0 | 86.0 | 55.3 | 55.6 | 55.4 |
| 10 | 36.7 | 88.0 | 53.5 | 53.6 | 53.9 |
| mean | 42.2 $\pm$ 3.7 | 80.3 $\pm$ 5.7 | 49.6 $\pm$ 4.7 | 49.5 $\pm$ 4.7 | 49.5 $\pm$ 4.7 |


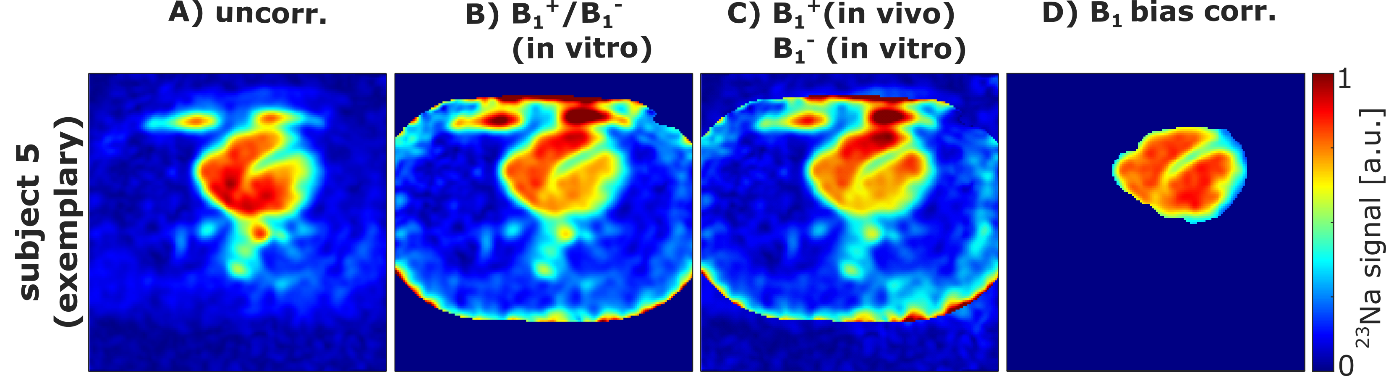


Figure S7: Influence of different B₁ correction methods on ²³Na MRI. The B₁ corrections were applied after respiratory and cardiac motion correction. Three different B₁ correction methods were compared: 1. B₁⁺ and B₁⁻ field measured in phantom; 2. B₁⁺ field measured in vivo using the AFI sequence and B₁⁻ field measured in phantom; 3: proposed B₁ bias field correction. Note that since the B_1_ bias field correction was applied only within the heart, the B_1_ bias corrected ^23^Na image was masked accordingly (D). ²³Na signal across the blood pool displayed a homogenous signal distribution for the B₁ bias field correction (D), which is not the case for the other two methods (B, C). Compared to the uncorrected image (A), B₁ bias field correction increased the ²³Na signal, in particular in superior left regions of the heart.


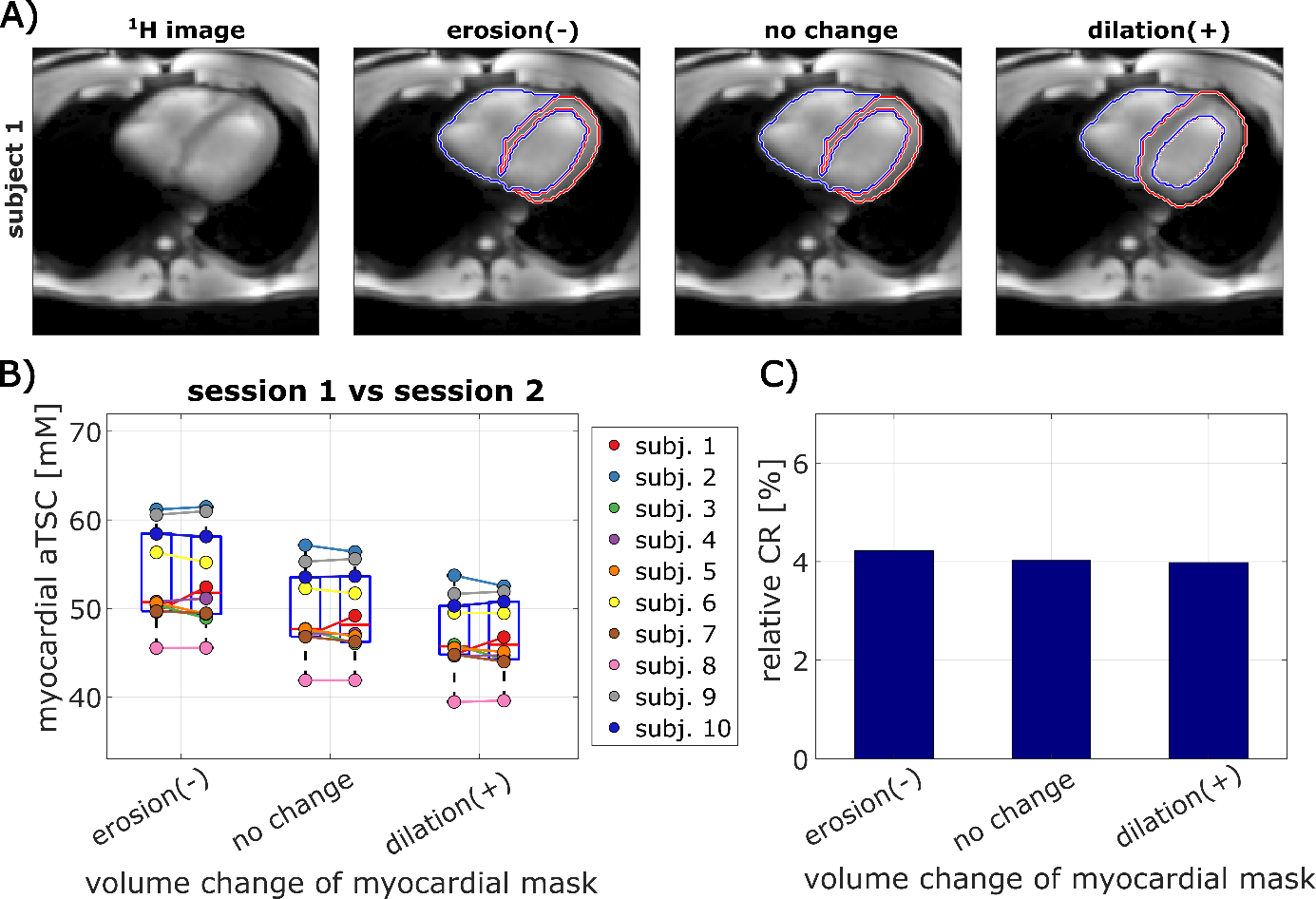


Figure S8: Influence of myocardial masks size on quantitative myocardial aTSC as well as repeatability. All corrections were applied as in Figure 10C, with B_1_ bias field correction and partial volume correction in particular depending on the segmentations. The original myocardial masks were eroded or dilated using a spherical structuring element with a radius of 1 voxel. The relative change in myocardial mask volume compared to the original mask, averaged across all ten subjects, was:–30% ± 3% for erosion and +30% ± 3% after dilation. **(A)** shows an overlay of the modified myocardial (red) and blood pool (blue) masks for subject 1. Reducing the myocardial mask size led to an increase in measured myocardial aTSC values, whereas dilation resulted in a decrease **(B).** The changes in global mean myocardial aTSC were: +7.6% for the eroded masks and –5.2% for the dilated masks. There were no relevant influences of the masks on the repeatability (C).


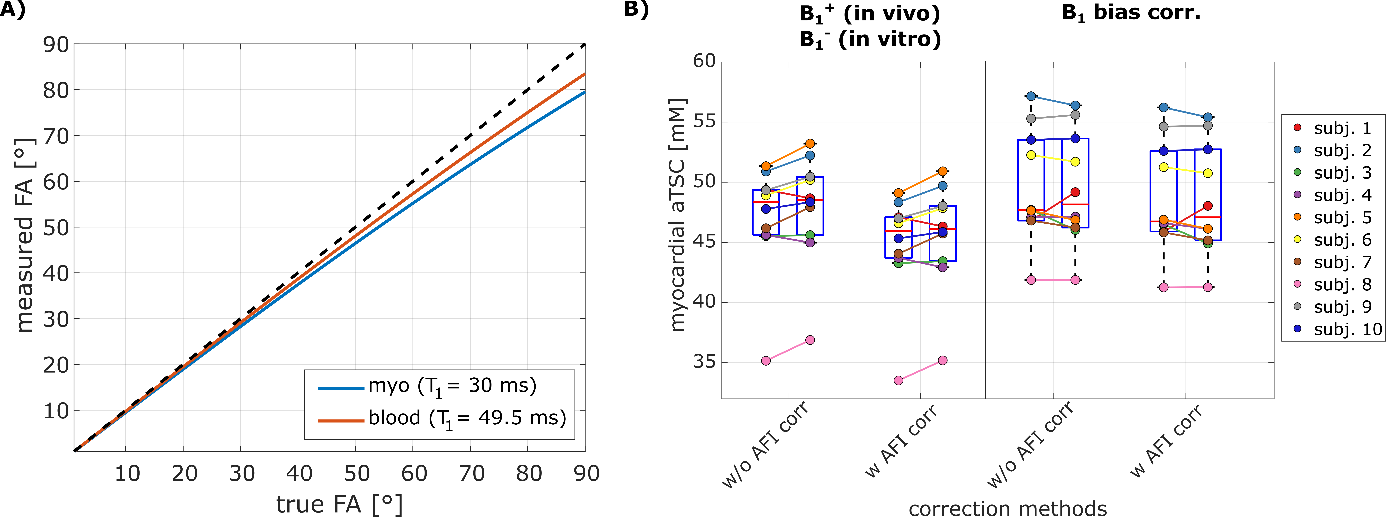


Figure S9: Influence of the AFI approximation on flip angles measured in myocardium and blood. The AFI method assumes $TR_{1/2}\ll T_{1}$. Since this is not fulfilled for the applied ^23^Na AFI measurements ($TR_{1}=12 ms, TR_{2}=48 ms$), this leads to deviations of the measured flip angle compared to the true flip angle (A). These deviations depend on the T_1_ relaxation time of the tissue and are therefore higher in myocardium than in blood. The found relation in A) allows to correct this effect for the different compartments assuming specific T_1_ times ($T_{1,myo}=30 ms, T_{1,blood}=49.5 ms$). For the method using the AFI for the estimation of the B_1_^+^ distribution and the B_1_^-^ map from the phantom (left side in B), this reduced the myocardial aTSC on average by 2.2mM and slightly worsened the repeatability from CR = 5.1% to 5.3%. When applying the B_1_ bias correction (right side in B), the AFI measurement was only used to determine the flip angle for the T_1_ relaxation correction. The average aTSC was decreased by -0.9mM, while the repeatability slightly improved from 4.0% to 3.9%.


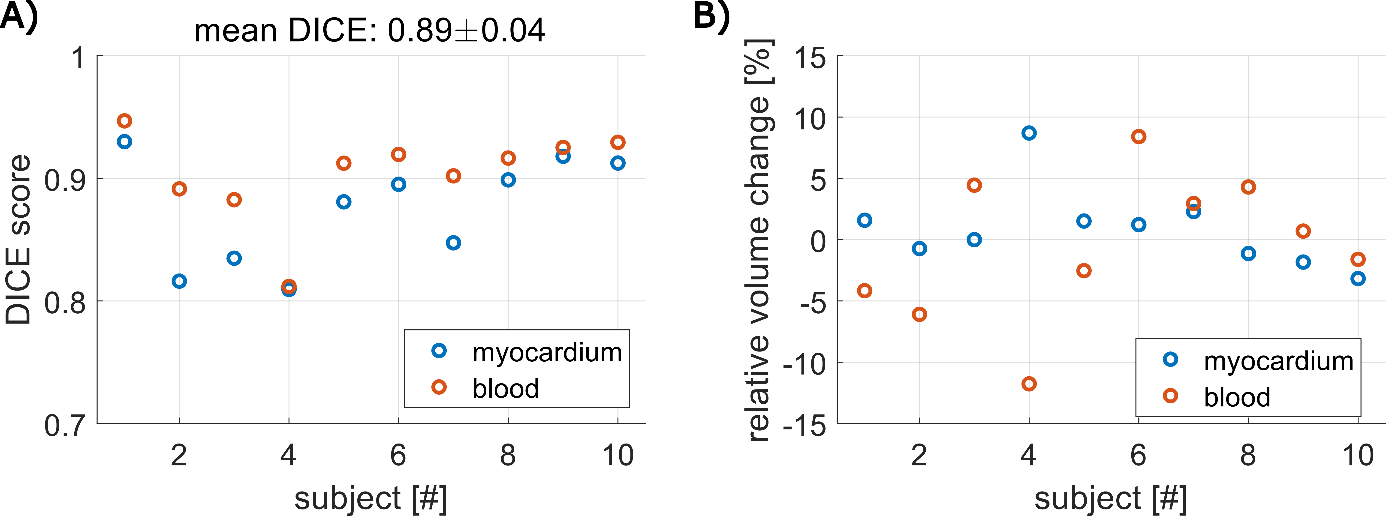


Figure S10: Assessment of the variability of the segmentations predicted by the nnUNet. For the evaluation of the DICE score the ^1^H image of the second measurement was registered to the ^1^H image of the first measurement. The segmentations of measurement 2 were than transformed accordingly. DICE scores for the myocardial and blood segmentation between both measurements (A) indicate good accordance. The mean DICE score across all subjects and both compartments was $0.89\pm0.04$. Relative volume changes of the myocardial and blood masks (B) ranged between -11.1% and 9.1%.


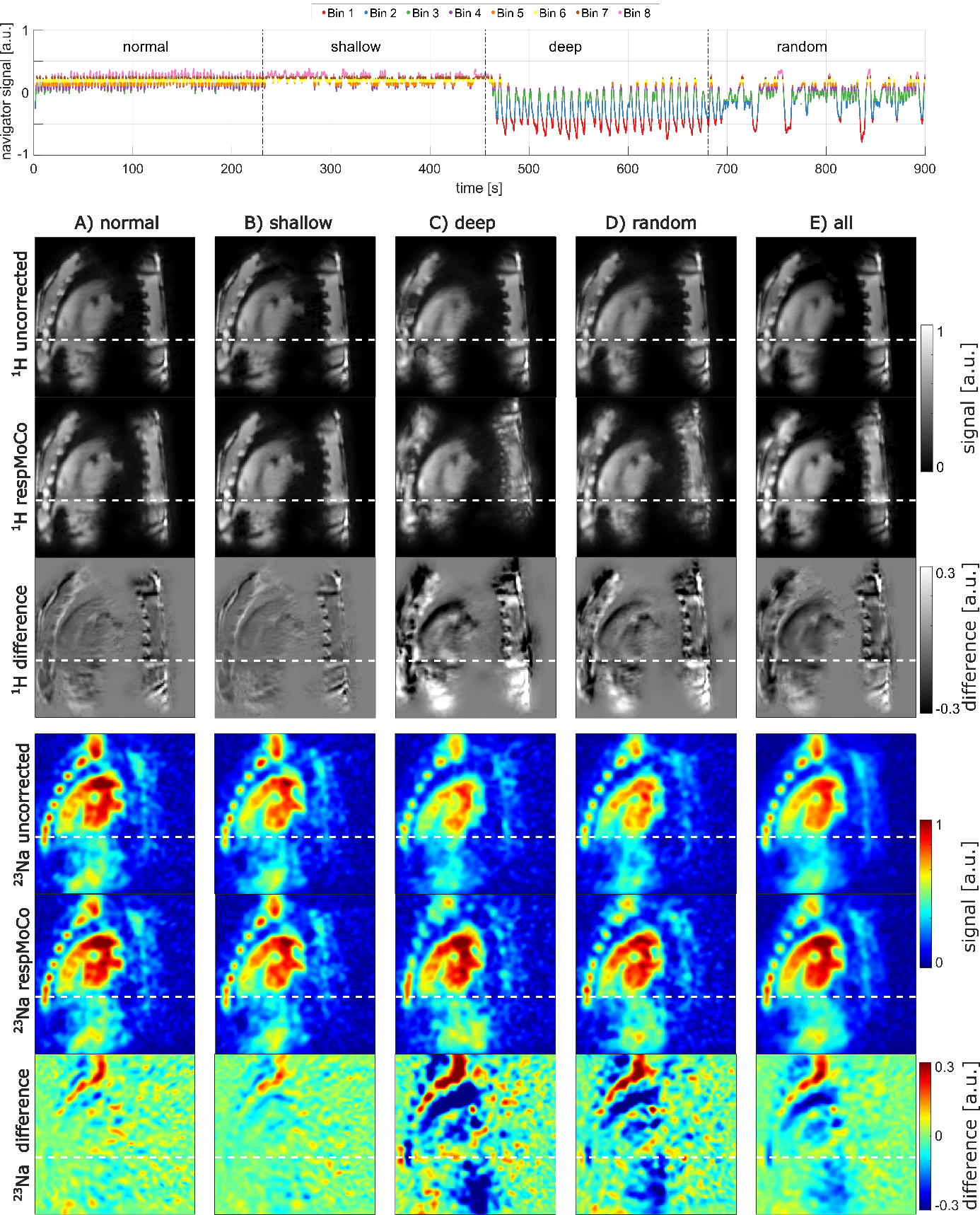


Figure S11: Validation of respiratory motion correction for different breathing patterns. In this measurement of one healthy subject (male, 28 years, 83 kg), the subject was instructed to change breathing patterns: A) normal breathing, B) shallow breathing, C) deep breathing, D) random breathing. Each pattern was performed for around 3:45 minutes. The ^1^H based self-gating navigator signal (top) captures all four breathing patterns and exhibits amplitude variations associated with different breathing depths. For the entire measurement the respiratory motion correction algorithm was applied as described in the paper, such that all acquired projections were binned into eight different respiratory bins and corrected into the fully-exhaled state. Here, bin 1 corresponds to the fully-inhaled state, while bin 8 represent the fully-exhaled state. For better assessment of the respiratory motion correction for the different breathing patterns, uncorrected and respiratory motion corrected ^23^Na and ^1^H images of each phase (A-D) were reconstructed as well as of the entire measurement (E). Note that the shown images do not contain cardiac motion correction and B_1_ correction was only applied for ^1^H, not ^23^Na MRI. Applying respiratory motion correction, ^23^Na and ^1^H images of all phases are aligned in the fully-exhaled state, while motion artifacts are especially visible in the uncorrected images for deep and random breathing. Differences between uncorrected and respiratory corrected images are more pronounced for deep and random breathing compared to normal or shallow breathing as expected.

# References

1. Bottomley PA. Sodium MRI in human heart: a review. *NMR in Biomed*. 2016;29(2):187–196.

2. Ouwerkerk R, Weiss RG, Bottomley PA. Measuring human cardiac tissue sodium concentrations using surface coils, adiabatic excitation, and twisted projection imaging with minimal T2 losses. *J Magn Reson Imaging*. 2005;21(5):546–555.

3. Lott J, Platt T, Niesporek SC, et al. Corrections of myocardial tissue sodium concentration measurements in human cardiac (23) Na MRI at 7 Tesla. *Magn Reson Med*. 2019;82(1):159–173.

4. James JR, Panda A, Lin C, Dydak U, Dale BM, Bansal N. In vivo sodium MR imaging of the abdomen at 3T. *Abdom Imaging*. 2015;40(7):2272–2280.

5. Zöllner FG, Konstandin S, Lommen J, et al. Quantitative sodium MRI of kidney. *NMR in Biomedicine*. 2016;29(2):197–205.

6. Kamp B, Frenken M, Klein-Schmeink L, et al. Evaluation of Sodium Relaxation Times and Concentrations in the Achilles Tendon Using MRI. *Int J Mol Sci* 2022;23(18):10890.

7. Madelin G, Jerschow A, Regatte RR. Sodium relaxation times in the knee joint in vivo at 7T. *NMR in Biomedicine*. 2012;25(4):530–537.

8. Schär M, Kozerke S, Fischer SE, Boesiger P. Cardiac SSFP imaging at 3 Tesla. *Magn Reson Med*. 2004;51(4):799–806.

9. Dobre MC, Uğurbil K, Marjanska M. Determination of blood longitudinal relaxation time (T1) at high magnetic field strengths. *Magnetic resonance imaging*. 2007;25(5):733–735.

10. Lin A-L, Qin Q, Zhao X, Duong TQ. Blood longitudinal (T 1) and transverse (T 2) relaxation time constants at 11.7 Tesla. *Magn Reson Mater Phys Biol Med*. 2012;25(3):245–249.

11. Rodgers CT, Piechnik SK, DelaBarre LJ, et al. Inversion recovery at 7 T in the human myocardium: measurement of T1, inversion efficiency and B1+. *Magn Reson Med*. 2013;70(4):1038–1046.

12. Huelnhagen T, Hezel F, Serradas Duarte T, et al. Myocardial effective transverse relaxation time Correlates with left ventricular wall thickness: A 7.0 T MRI study. *Magn Reson Med*. 2017;77(6):2381–2389.

13. Ren J, Dimitrov I, Sherry A, Malloy C. T1 and T2 relaxation time measurements of metabolites in human calf muscle at 7 Tesla. In Proceedings of the 17th Annual Meeting of ISMRM, Honolulu, Hawaii, 2009. 1915.

14. Li X, Bolan PJ, Ugurbil K, Metzger GJ. Measuring renal tissue relaxation times at 7 T. *NMR in Biomed*. 2015;28(1):63–69.

15. Lazik A, Theysohn JM, Geis C, et al. 7 Tesla quantitative hip MRI: T1, T2 and T2* mapping of hip cartilage in healthy volunteers. *Eur Radiol*. 2016;26(5):1245–1253.

16. Gast LV, Platt T, Nagel AM, Gerhalter T. Recent technical developments and clinical research-applications of sodium (23Na) MRI. *Prog Nucl Magn Reson Spectrosc*. 2023.
